# Structural Transformation of Metastable Two-Electron Superatom Au-Doped Cu-Rich Alloy Nanocluster

**DOI:** 10.3390/molecules29184427

**Published:** 2024-09-18

**Authors:** Rhone P. Brocha Silalahi, Samia Kahlal, Jean-Yves Saillard, C. W. Liu

**Affiliations:** 1 Department of Chemistry, National Dong Hwa University, Hualien 97401, Taiwan; rhone.p.brocha.silalahi@gmail.com; 2 Univ Rennes, CNRS, Institut des Sciences Chimiques de Rennes-UMR 6226, F-35000 Rennes, France; kahlal@univ-rennes1.fr

**Keywords:** copper, gold, hydride, superatom, structural transformation

## Abstract

The ability to fabricate bimetallic clusters with atomic precision offers promising prospects for elucidating the correlations between their structures and properties. Nevertheless, achieving precise control at the atomic level in the production of clusters, including the quantity of dopant, characteristic of ligands, charge state of precursors, and structural transformation, have remained a challenge. Herein, we report the synthesis, purification, and characterization of a new bimetallic hydride cluster, [AuCu_11_(H){S_2_P(O*^i^*Pr)_2_}_6_(C≡CPh)_3_] (**AuCu_11_H**). The hydride position in **AuCu_11_H** was determined using DFT calculations. **AuCu_11_H** comprises a ligand-stabilized defective *fcc* Au@Cu_11_ cuboctahedron. **AuCu_11_H** is metastable and undergoes a spontaneous transformation through ligand exchange into the isostructural [AuCu_11_(Cl){S_2_P(O*^i^*Pr)_2_}_6_(C≡CPh)_3_] (**AuCu_11_Cl**) and into the complete cuboctahedral [AuCu_12_{S_2_P(O*^i^*Pr)_2_}_6_(C≡CPh)_4_]^+^ (**AuCu_12_**) through an increase in nuclearity. These structural transformations were tracked by NMR and mass spectrometry.

## 1. Introduction

Structural transformation in atomically precise metal nanoclusters (NCs) is important not only to investigate their structure–property relationships but also to serve as a unique platform for exploring structural evolution [1,2,3]. In the past decade, research in the field of structural NC transformations has broadened significantly, and many NCs resulting from structural evolutions have been synthesized [4,5,6]. The structural transformation of the metal core process can occur in certain metal NCs under suitable conditions, such as redox treatment [7,8], pH induction [9,10], ligand exchange [11,12], and elevated temperatures [13,14,15,16,17]. It can lead to the formation of structural isomers of various sizes [4,5,6,7,8,9,10,11,12,13,14,15,16,17,18]. While the structural transformation of pure and alloyed gold and silver clusters has been extensively studied, there has been limited research on their copper homologues [19,20,21,22,23,24,25,26,27,28,29,30,31,32]. A noticeable example, reported by Zhu et al., is the PPh_3_ addition to [Cu_41_(SC_6_H_3_F_2_)_15_(P(PhF)_3_)_6_C_l3_H_25_]^2−^ that induces the conversion into [Cu_14_(SC_6_H_3_F_2_)_3_(PPh_3_)_8_H_10_]^+^, while the addition of P(PhF)_3_ converts it into [Cu_13_(SC_6_H_3_F_2_)_3_(P(PhF)_3_)_7_H_10_] [33]. Another remarkable example reported by Zang et al. is the step-by-step controlled preparation of two catalytically active carborane alkynyl-protected copper NCs, namely [Cu_13_(C_4_B_10_H_11_)_10_(PPh_3_)_2_(CH_3_CN)_2_]^3+^ and [Cu_26_(C_4_B_10_H_11_)_16_(C_2_B_9_H_10_C_2_)_2_(PPh_3_)_2_(CH_3_CN)_4_]^4+^, through external ligand shell modifications and metal-core evolution [34]. We also reported the structural transformation under acidic conditions of the cuboctahedral [Cu_12_S{S_2_CN*^n^*Bu}_2_(C_2_Ph)_4_] into the fused bi-cuboctahedral [Cu_21_S_2_{S_2_CN*^n^*Bu_2_}_9_(C_2_Ph)_6_] [35] and the spontaneous transformation in a chlorinated solvent of [Cu_15_H_2_{S_2_CN*^n^*Bu_2_}_6_(C_2_Ph)_4_]^+^ into the [Cu_13_{S_2_CN*^n^*Bu_2_}_6_(C_2_Ph)_4_]^+^ two-electron superatom [36].

It is to be noted that research on NC transformation has generally focused on stable species, while the analysis of the structural evolution of metastable NCs has only begun to receive attention in the last few years [37,38,39,40,41]. Capturing metastable clusters with a labile nature provides valuable information for understanding the pathways of metal NC transformations [42]. From this point of view, hydride-stabilized NCs constitute a privileged field of investigations [25,26,27,28,29,30,31,37,38,39,40,41,42,43,44,45,46,47,48,49]. Indeed, the coordination diversity and H-dissociation behavior in hydride-ligated NCs make them a dynamic system that can facilitate structural modifications [47]. The relatively weak M-H bond allows for the easy substitution of hydrides by other ligands, which is an effective way to obtain the evolution of intermediates [47,49]. In this context, by introducing a hydride ligand on two-electron superatoms, Au-Cu alloy NCs, the structural transformation pathway is expected to be particularly studied at the simplified hydride-binding site. To the best of our knowledge, no two-electron superatom Au-doped Cu-rich hydride has been reported so far, and hydride ligands are still rarely observed in alloy nanoclusters [50]. This work aims to obtain the isostructural Au-Cu bimetallic cluster and acquire insight into the underlying mechanism of transformation of a two-electron superatom, thereby enhancing its nuclearity.

## 2. Results and Discussion

**Scheme 1:**
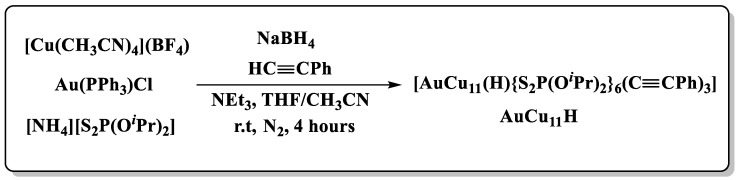
Synthesis route for **AuCu_11_H**.

**AuCu_11_H** was first prepared by a one-pot procedure with mixed solvent THF-CH_3_CN (1:1). The synthetic procedure of **AuCu_11_H** differs from that reported in **AuCu_11_Cl** [25] and **AuCu_12_** [26]. The synthesis protocol involves the reduction of copper and gold precursors with the reducing agent NaBH_4_ in the presence of alkynyl, trimethylamine, and dithiophosphate (dtp) ligands (Scheme 1). The yellowish-green mixture immediately turned yellow after addition of the reducing agent and finally dark brown after stirring for 4 h at ambient temperature. The resulting mixture was evaporated by a rotavapor to give a dark brown solid. This solid was purified by an aluminum oxide column to afford an orange-red precipitate of **AuCu_11_H** in 13% yield.

**Figure 1:**
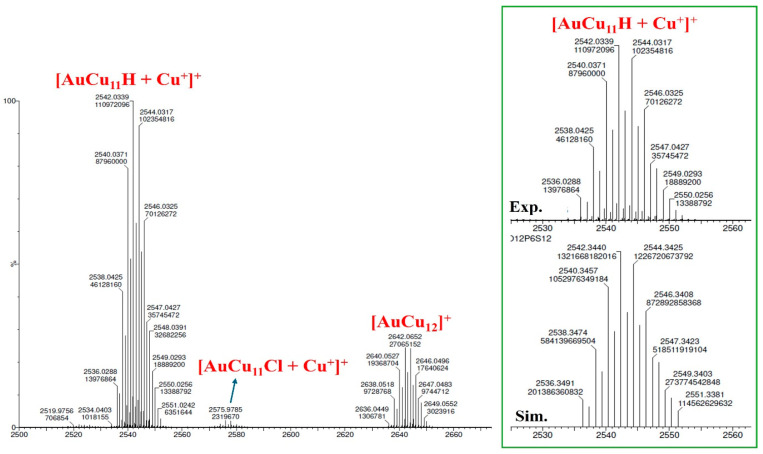
ESI-MS spectra of [**AuCu_11_H** + Cu^+^]^+^. The inset shows the comparison between the experimental data (**top**) and the simulated (**bottom**) isotope patterns for [**AuCu_11_H** + Cu^+^]^+^.

The deuterated derivatives [AuCu_11_(D){S_2_P(O*^i^*Pr)_2_}_6_(C≡CPh)_3_] (**AuCu_11_D**) were also synthesized to support the existence of the hydride in the clusters. Synthetic details are provided in the supporting information. Electrospray ionization mass spectrometry (ESI-MS) in a positive mode was performed to verify the molecular formula of **AuCu_11_H**. The ESI-MS spectra of **AuCu_11_H** (Figure 1 and Table 1) show two prominent peaks at *m*/*z* = 2542.03 and 2642.37 Da, which correspond to the molecular weight of **AuCu_11_H** with an adduct of one copper ion, [AuCu_11_(H){S_2_P(O*^i^*Pr)_2_}_6_(C≡CPh)_3_ + Cu^+^]^+^ (*m*/*z* calc. = 2542.34 Da), and the molecular ion [AuCu_12_{S_2_P(O*^i^*Pr)_2_}_6_(C≡CPh)_4_]^+^ (*m*/*z* calc. = 2642.37 Da), respectively. The smaller peak can be ascribed to [AuCu_11_(Cl){S_2_P(O*^i^*Pr)_2_}_6_(C≡CPh)_3_ + Cu^+^]^+^ at *m*/*z* = 2575.96 Da (*m*/*z* calc. = 2575.31 Da). The measured isotopic pattern fully matches with the simulated pattern (Figure 1 (inset) and Figure S1). To confirm the number of hydride(s), a controlled preparation with NaBD_4_ was performed to obtain the deuterated **AuCu_11_D**. The ESI-MS analysis of **AuCu_11_D** depicts two intense peaks at *m*/*z* = 2543.37 Da (*m*/*z* calc. = 2543.35 Da) and 2478.43 Da (*m*/*z* calc. = 2478.41 Da) (Figure S2), which correspond to the molecular weight of **AuCu_11_D** with a copper adduct ion, [AuCu_11_(D){S_2_P(O*^i^*Pr)_2_}_6_(C≡CPh)_3_ + Cu^+^]^+^, and neutral [AuCu_11_(D){S_2_P(O*^i^*Pr)_2_}_6_(C≡CPh)_3_], respectively. The mass spectra also display the molecular ion [AuCu_12_{S_2_P(O*^i^*Pr)_2_}_6_(C≡CPh)_4_]^+^ at *m*/*z* = 2642.40 Da (*m*/*z* calc. = 2642.37 Da) (Figure S2). A distinct shift of *m*/*z* = 1.34 is observed between the mass of **AuCu_11_H** and **AuCu_11_D**, confirming that the total number of hydrides in **AuCu_11_H** is one. The theoretically determined isotopic patterns of [**AuCu_11_D** + Cu^+^]^+^, [**AuCu_11_D**], and [**AuCu_12_**]^+^ show excellent agreement with the experiment, as depicted in Figures S2 (inset) and S3, respectively.

**Figure 2:**
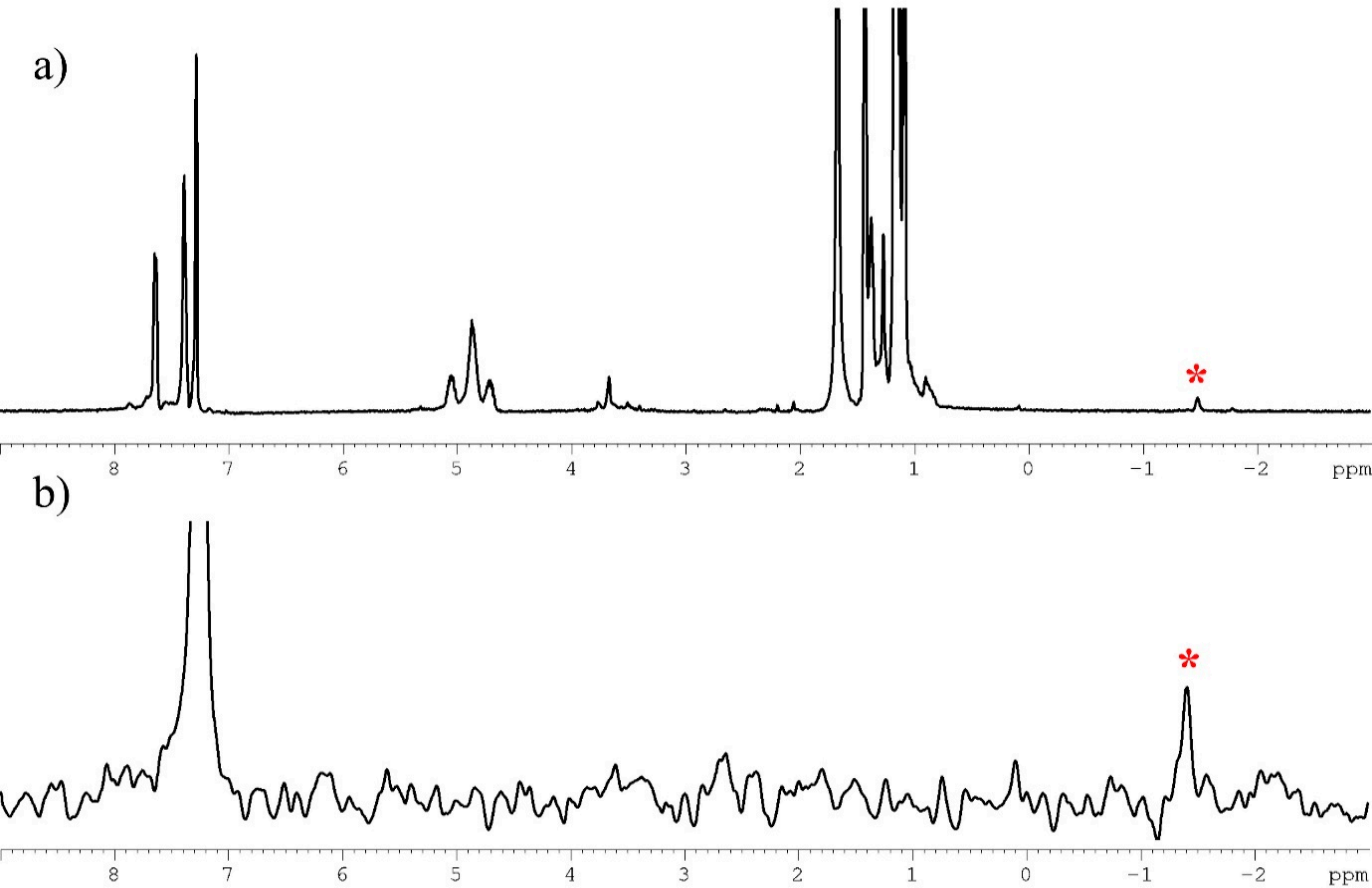
(**a**) The ^1^H NMR spectrum of **AuCu_11_H** in CDCl_3_. (**b**) The ^2^H NMR spectrum of **AuCu_11_H** in CHCl_3_. The asterisk symbol signifies the hydride peak.

The ^1^H NMR spectrum of **AuCu_11_H** is shown in Figure 2. The presence of the hydride is confirmed with a chemical shift at –1.49 ppm (Figure 2a). The peak integration ratios indicate six dtp and three alkynyl ligands to one hydride. The signals at 4.69–5.06 and 1.07–1.43 ppm in a 1:6 intensity ratio are attributed to the -OCH and CH_3_ protons of the dtp isopropyl groups, respectively (Figure 2a). In addition to the isopropyl peaks, the spectrum also shows the two peaks of the alkynyl protons at 7.39–7.64 ppm. The corresponding deuterated resonance in the ^2^H NMR spectra of **AuCu_11_D** confirms the position of the hydride with relatively similar resonances observed at −1.41 ppm. (Figure 2b and Figure S4). The ^31^P{^1^H} spectrum of **AuCu_11_H** displays three signals at 104.4, 100.5, and 99.9 ppm attributed to three types of di-isopropyl dithiophosphate ligands in a symmetrical structure **AuCu_11_H** (Figures S5 and S6). In the ^13^C NMR spectrum, the peaks at 23.2 and 77.2 ppm are assigned to the isopropyl group of dithiophosphates, followed by the peaks at 125.2, 128.9, 132.3, 134.1, and 77.2 and 137.4 ppm corresponding to the aromatic carbons of the phenyl rings and the carbon atoms of the C≡C functional groups, respectively (Figure S7). A similar synthetic procedure was conducted to obtain two derivatives of A**uCu_11_H** by using di-n-propyl and di-isobutyl dithiophosphate ligands and 4-ethylanisole. All their spectroscopic data are consistent with the proposed formula (see Materials and Methods section, Figures S8–S20).

**Figure 3:**
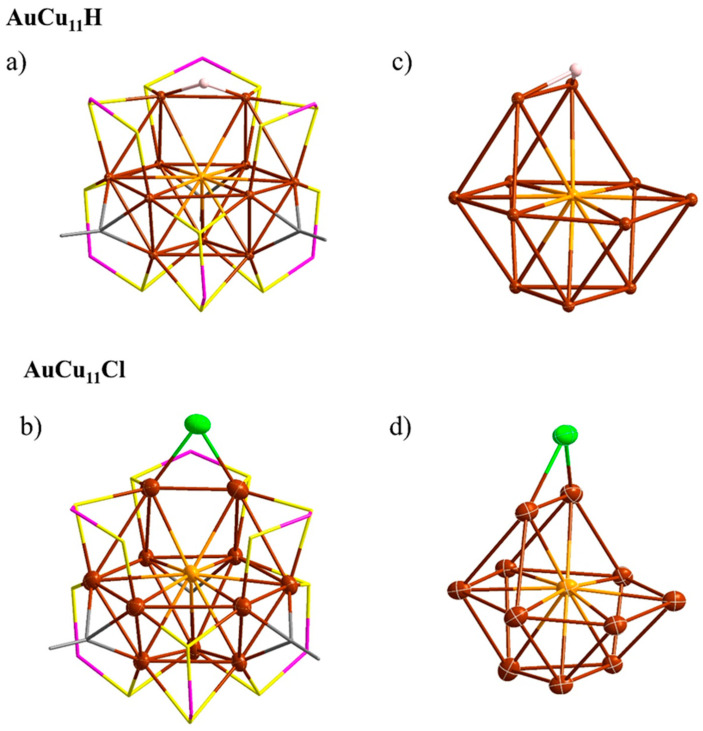
The DFT-optimized structure of **AuCu_11_H** and the X-ray structure of **AuCu_11_Cl** [41]: (**a**,**b**) overall structures; (**c**,**d**) the Au@Cu_11_ cuboctahedral core with the bridging hydride–chloride ligand. All the phenyl rings of alkynyls and the alkyl group of dtp ligands are omitted for clarity. Color labels: dark red = Cu; orange = Au; yellow = S; magenta = P; pink = H; green = Cl, gray = C.

Due to the inherent instability of **AuCu_11_H** in solution, it was not possible to obtain a single crystal of **AuCu_11_H**. Nevertheless, a comparison of the NMR data collected for this compound with those of the isoelectronic **AuCu_11_Cl** [25] suggests strongly that both NCs have a similar structure. This hypothesis is also supported by the fact that the composition of the two compounds differs only by the nature of one ligand (H vs. Cl). Then, a geometry optimization of **AuCu_11_H** by means of density functional theory (DFT) at the PBE0/Def2-TZVP was performed (see computational details), starting from that of the **AuCu_11_Cl** parent, and indeed the converged structure presented similar features as its chloride homologue. Other structural arrangements were also tested, including several with different hydride positions on different metal atoms (including Au) and in different coordination modes, but the global minimum was found to remain definitely the same, i.e., both **AuCu_11_H** and **AuCu_11_Cl** are isostructural, including with respect to the location of the H vs. Cl ligand (see Figure 3). The secondary minimum of lowest energy was found to be at a much higher energy (ΔE = 1.40 eV) to allow considering any equilibrium in solution. In this isomer, the hydride is shifted on the opposite side of the vacancy and bridges an AuCu_2_ triangle (see SI). The AuCu_11_ core describes an Au-centered cuboctahedron of which one vertex is missing. This defective *fcc* Au@Cu_11_ core is decorated with four (µ_2_, µ_2_) and two (µ_2_, µ_1_) dtp ligands and three (µ_3_-η^1^) alkynyl ligands (Figure 3a,b). The hydride bridges one of the Cu-Cu edges, which borders the core vacancy. This location makes sense in the way that the hydride (or chloride in the case of **AuCu_11_Cl**) is linked to the two copper atoms that would be the least connected metals if this ligand was missing. It is noteworthy that the hydride ligand in **AuCu_11_H** becomes closer to the central gold atom than the chloride in **AuCu_11_Cl** (Figure 3c,d). Nevertheless, the Au…H distance (2.851 Å) is of the order of magnitude of a van der Waals contact, whereas the corresponding Cu-H distances of **AuCu_11_H** (1.633 Å) correspond to standard Cu-(µ_2_-H) bonds. Other average computed metrical data are provided in Table 2. They are comparable to those previously calculated for **AuCu_11_Cl** [25], also reported in Table 2 for comparison.

The calculated ^1^H NMR chemical shift (see SI for computational details) of the hydride (1.3 ppm) is in a satisfying agreement with the experimental value (−1.49 ppm), owing to the fact that a discrepancy of ~3 ppm is not uncommon for metal-bound hydrides in these types of clusters [51]. The computed chemical shifts of the ligand protons match well with the experiment, with averaged value of 7.3, 7.4, and 7.8 ppm (aromatic protons), 4.8 ppm (-OCH), and 0.8 ppm (CH_3_), as well as the ^13^C chemical shifts (88.4 and 140.4 (C≡C), 135.6, 128.4, 127.6, and 126.5 (aromatic carbons), and 70.3 and 20.2 (isopropyl carbons)).

**Figure 4:**
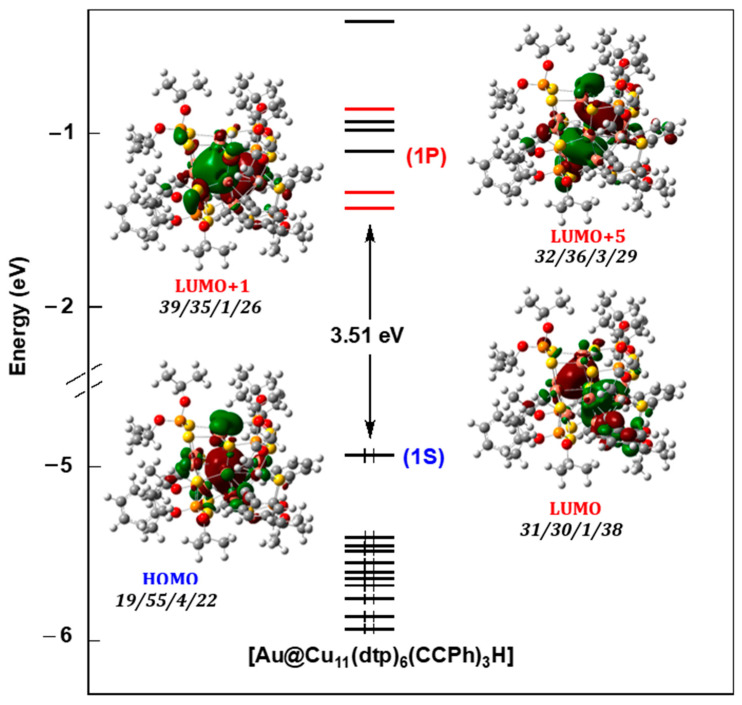
The Kohn-Sham frontier orbital diagram of **AuCu_11_H**. The orbital contribution (in %) is given in the order Au/Cu_11_/H/ligands.

The KohnSham orbital diagram of **AuCu_11_H** (Figure 4) is typical of the two-electron superatoms based on a centered complete (*closo*-type) [52] or defective (*nido*-type) [25] cuboctahedron. It is characterized by a 1S HOMO with a dominant Au character. A peculiarity of this diagram is the fact that only two among the 1P orbitals are the lowest unoccupied levels, the third one being the LUMO+5. The somewhat higher energy of the latter could be due to some antibonding (weak) hydride admixture.

**Figure 5:**
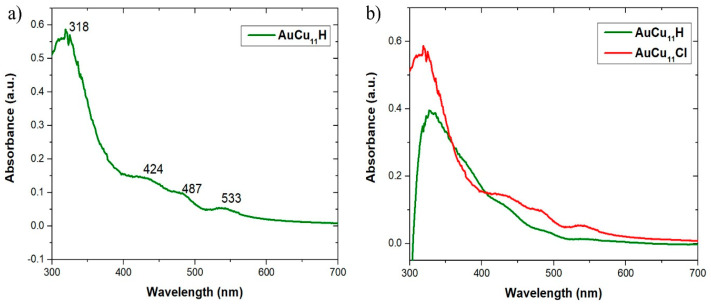
(**a**) The experimental UV–vis absorption spectrum of **AuCu_11_H** in MeTHF. (**b**) Comparison of UV-vis absorption spectra of **AuCu_11_H** and **AuCu_11_Cl** in MeTHF.

The UV-vis spectrum of **AuCu_11_H** is shown in Figure 5a. There are three weak bands at 424, 487, and 533 nm, and another highly structured one at 318 nm. Figure 5b compares the UV-vis absorption spectra of **AuCu_11_H** and **AuCu_11_Cl**. **AuCu_11_Cl** also displays four absorption peaks at 328, 432, 485, and 540 nm [25]. These similarities in the UV-vis spectra also support the fact that both NCs are isostructural. Their multiband absorption profiles are typical of superatom-type clusters (Figure S21) [25,26,29]. The TD-DFT-simulated spectrum of **AuCu_11_H** is shown in Figure S22. Owing to the shapeless nature of its experimental counterpart, the agreement is satisfying. A similar agreement was found for **AuCu_11_Cl** [25]. The broad band of lowest energy results from a combination of HOMO→LUMO and HOMO→LUMO+1 transitions; hence, it is of superatomic 1S→1P nature, whereas the high-intensity peak at high energy (322 nm) is found to be of MLCT character, resulting from transitions between Cu(3d) levels and π*(phenyl) combinations.

**Figure 6:**
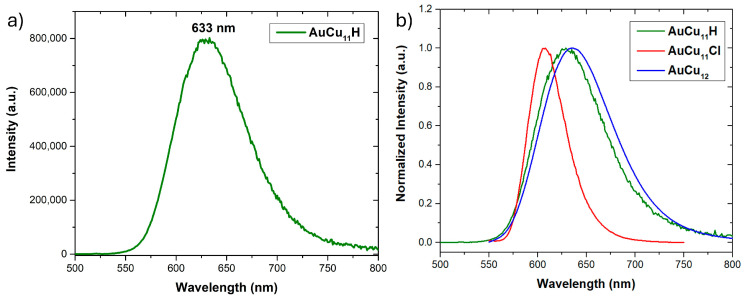
(**a**) The emission spectrum of **AuCu_11_H**. (**b**) Comparison emission spectra of **AuCu_11_H**, **AuCu_11_Cl**, and **AuCu_12_** in MeTHF at ambient temperature.

The photoluminescence (PL) properties of the twoelectron superatomic **AuCu_11_H** and **AuCu_11_Cl** relatives were analyzed and compared (Figure 6). Both NCs are emissive at ambient temperatures in MeTHF. The excitation spectrum of **AuCu_11_H**, shown in Figure S23, is closely related to its absorption UV-vis spectrum. Upon exciting at 437 nm, **AuCu_11_H** (in MeTHF) emits at 633 nm, with a PL quantum yield (QY) of 32.7%. On the other hand, **AuCu_11_Cl** emits at 606 nm, with a ten times lower PL QY of 3.3% [25]. Interestingly, the emission band of **AuCu_11_H** is close to that of **AuCu_12_** at 637 nm, with a QY of 32% [26]. Thus, the PL QY for the two-electron Au-doped Cu-rich alloy clusters follows the order **AuCu_12_** > **AuCu_11_H** > **AuCu_11_Cl**. The PL lifetime of **AuCu_11_H** is 1.71 μs (Figure S24), whereas that of **AuCu_11_Cl** and **AuCu_12_** were determined to be 3.75 and 3.20 μs. Such a PL lifetime quenching is attributed to the hydride-chloride exchange and reveals the instability of **AuCu_11_H** in solution. Nevertheless, the high QY of **AuCu_11_H** (*k*_rad_ = 0.19 μs^−1^) might originate from a similar value of the radiative rate constant as **AuCu_12_** (*k*_rad_ = 0.10 μs^−1^) (Table S1) [25,26].

**Scheme 2:**
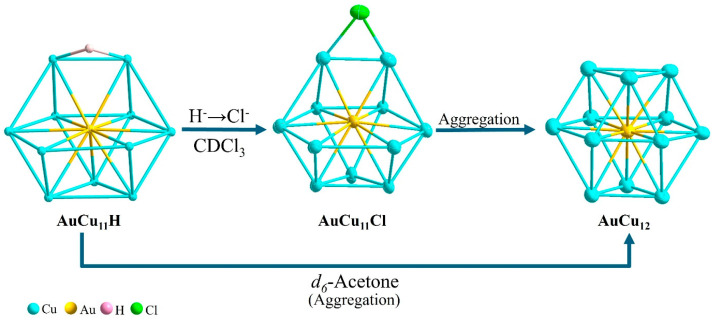
Structural transformation of **AuCu_11_H** to **AuCu_12_** via **AuCu_11_Cl** in solution.

**AuCu_11_H** is metastable in solution, spontaneously converting into **AuCu_11_Cl** and **AuCu_12_** (Scheme 2). The transformation process was monitored by both time-dependent NMR spectroscopy in different types of solvents and ESI-MS analysis. A non-chlorinated solvent was first employed for the investigation. As shown in Scheme 2, a direct conversion of **AuCu_11_H** into **AuCu_12_** occurs in acetone. The ^1^H NMR chemical shift of the isopropyl groups in **AuCu_11_H** gradually merges with time and shows good agreement with the chemical shift of the isopropyl group in **AuCu_12_** (Figures S25 and S26). No hydride peak can be identified in the ^1^H NMR spectrum after 24 h. Moreover, whereas the time-dependent ^31^P{^1^H} NMR spectra of **AuCu_11_H** exhibited single sharp peaks at 104.4, 100.5, and 99.9 ppm, respectively, the sharp resonances at 104.4 and 99.9 coalesced into a single sharp peak of 100.5 ppm, which correlates to the ^31^P{^1^H} chemical shift of **AuCu_12_** (Figures S27 and S28). This is also supported by the ESI-MS spectrometry, as depicted in Figures S29 and S30. Thus, during the conversion, **AuCu_11_H** undergoes aggregation and is accompanied by the rearrangement of the core structure from a vacancy defect Au@Cu_11_ cuboctahedron to a full **Au@Cu_1_**_2_ cuboctahedron after the successive addition of one copper acetylide unit.

In contrast, the conversion of **AuCu_11_H** in a chlorinated solvent leads to the formation of **AuCu_12_** via **AuCu_11_Cl** (Scheme 2). The ^1^H NMR spectra identified that the hydride peak gradually vanished after 48 h (Figure S31). As shown in Figure S32, the intensity of the ^31^P{^1^H} peaks of **AuCu_11_H** at 104.4 and 99.9 ppm decreases with time, followed by a new peak at 97.8 ppm, which corresponds to the ^31^P{^1^H} chemical shift of **AuCu_11_Cl** (Figure S33). These time-dependent spectra suggest that the presence of the chloride ion from the chlorinated solvent triggered the hydride-chloride exchange, and the hydride tends to dissociate, releasing H^+^ and being replaced by the chloride ion [25]. Hydride and chloride exchange slowly, giving rise to a weak **AuCu_11_Cl** signal at *m*/*z* = 2578.32 Da (*m*/*z* calc = 2578.30 Da) in the mass spectrum (Figures S34 and S35). Furthermore, this transformation is followed by the degradation of **AuCu_11_Cl** to **AuCu_12_** through a partial aggregation process [25]. These results are supported by the ^31^P{^1^H} NMR resonance at 97.8 ppm, which shifts to 100.7 ppm within 96 h, i.e., to the chemical shift of **AuCu_12_**. We thus propose that transformation in the chlorinated solvent allows the metastable **AuCu_11_H** to be the key precursor in the formation of **AuCu_12_** through **AuCu_11_Cl**. The time-dependent ^1^H NMR of **AuCu_11_H** in the chlorinated solvent shows the characteristic of a hydride ligand, indicating less stability of **AuCu_11_H** in the solution. Based on this result, the hydride in **AuCu_11_H** acts as an electron-withdrawing hydride, as shown by the dissociation of H^−^ and subsequently followed by the replacement of the chloride ligand to generate **AuCu_11_Cl**. In addition, **AuCu_11_Cl** grows via partial aggregation to form **AuCu_12_** following the sequential addition of one copper acetylide unit (Scheme S1). This study shows that Au-Cu bimetallic NCs grow by creating a hydride bimetallic complex and then exchanging ligands with H^−^ dissociation.

## 3. Materials and Methods

### 3.1. General Remarks

All chemicals were used without further purification and purchased from commercial sources as follows: phenylacetylene, sodium borohydride, sodium borodeuteride, and all the required anhydrous solvents were purchased from Sigma Aldrich. All reactions were conducted in a N_2_ atmosphere utilizing typical Schlenk procedures. [Cu(CH_3_CN)_4_](PF_6_) [53], Au(PPh_3_)Cl [54], and [NH_4_][S_2_P(OR)_2_] (R = *^i^*Pr, *^n^*Pr, and *^i^*Bu) [55] were synthesized according to the methodology documented in the literature and then described. NMR spectra were acquired using a Bruker Advance DPX300 FT-NMR spectrometer running at 400 MHz for ^1^H, 121.5 MHz for ^31^P{^1^H}, 100.61 MHz for ^13^C{^1^H}, and 46.1 MHz for ^2^H. The ^1^H NMR chemical shift was referenced to residual CDCl_3_ (δ = 7.26 ppm) and deuterated acetone (δ = 2.04 ppm). The ^13^C{^1^H} NMR spectra were referenced to deuterated acetone (δ = 29.8 and 206.3 ppm). The ^31^P{^1^H} NMR spectra were referenced to external 85% H_3_PO_4_ at δ = 0 ppm. The chemical shift (δ) is expressed in ppm, and processing was carried out with Bruker’s Topspin 2.1 software. The ESI-mass spectrometry was obtained using a Fison Quattro Bio-Q (Fisons Instruments, VG Biotech, U.K.). The UV–visible absorption spectra were acquired using a Perkin Elmer Lambda 750 spectrophotometer with quartz cells featuring a 1 cm path length. An Edinburgh FLS920 spectrophotometer captured the photoluminescence excitation and emission spectra using the Xe900 lamp as the excitation source. The PL decays were assessed utilizing an Edinburgh FLS920 spectrometer equipped with a gated hydrogen arc lamp and a scattering solution to characterize the instrument response function.

### 3.2. General Synthesis

The clusters [AuCu_11_(H){S_2_P(O*^i^*Pr)_2_}_6_(C≡CPh)_3_], [AuCu_11_(H){S_2_P(O*^n^*Pr)_2_}_6_(C≡CPh)_3_], and [AuCu_11_(H){S_2_P(O*^i^*Bu)_2_}_6_(C≡CPh)_3_] can be prepared by following general procedures, and a detailed preparation method is given for cluster [AuCu_11_(H){S_2_P(O*^i^*Pr)_2_}_6_(C≡CPh)_3_] (**AuCu_11_H**).

#### 3.2.1. Synthesis of [AuCu_11_(H){S_2_P(O^i^Pr)_2_}_6_(C≡CPh)_3_] (**AuCu_11_H**)

In a Flame-dried Schlenk tube [NH_4_][S_2_P{O*^i^*Pr}_2_] (30 mg, 0.15 mmol), Cu(CH_3_CN)_4_](BF_4_) (130 mg, 0.30 mmol) and Au(PPh_3_)Cl (4 mg, 0.1 mmol) were suspended in a mixture of THF (15 cm^3^) and CH_3_CN (15 cm^3^) followed by the continuous addition of phenylacetylene (40 µL, 0.3 mmol) and triethylamine (40 µL, 0.3 mmol). After stirring for 5 min, NaBH_4_ (6 mg, 0.15 mmol) was added to the mixture. The resulting mixture was stirred at room temperature for 4 h. The solvent was evaporated under vacuum, and the residue was dissolved in DCM and washed with water (3 × 15 mL). After separation, the organic layer was passed through aluminum oxide, and the orange-red residue was washed with ether and ethyl acetate to get rid of the impurities. The precipitate was then dissolved in methanol (3 × 15 mL) to collect the orange-red solution. Finally, the solvent was evaporated to dryness under vacuum to obtain a pure orange-red powder of **AuCu_11_H**. The yield was 13% based on Cu.

Similarly, the deuterium analog [AuCu_11_(D){S_2_P(O*^i^*Pr)_2_}_6_(C≡CPh)_3_] was synthesized by reacting NaBD_4_ (0.0042, 0.15 mmol), resulting in the formation of [AuCu_11_(D){S_2_P(O*^i^*Pr)_2_}_6_(C≡CPh)_3_] (15%, based on Cu).

[**AuCu_11_H**]: ESI-MS: *m*/*z* 2542.37 Da (calcd. *m*/*z* 2542.34 Da) for [AuCu_11_(H){S_2_P(O*^i^*Pr)_2_}_6_(C≡CPh)_3_ + Cu^+^]^+^. ^1^H NMR (400 MHz, CDCl_3_): 7.39 (8H, d, C_6_*H*_5_), 7.64 (7H, d, C_6_*H*_5_), 4.69–5.06 (12H, m, OC*H*), 1.07–1.43 (72H, dd, C*H*_3_), and −1.49 (1H, s, μ_2_-H) ppm. ^31^P NMR (121.5 MHz, CDCl_3_): 104.45, 100.49, and 99.90 ppm. ^13^C NMR (100.61 MHz, *d*_6_-acetone): 205.33 (CH_3_***C***OCH_3_), 29.84 (***C***H_3_CO***C***H_3_), 134.08 (*o*-***C***_6_H_6_), 132.27 (*o*-***C***_6_H_6_), 128.85 (*p*-***C***_6_H_6_), 125.17 (*p*-***C***_6_H_6_), 77.80 (***C***≡C), 76.18 (C≡***C***), 34.13 (-O***C***H(CH_3_)_2_), and 23.22 (-OCH(***C***H_3_)_2_).

[**AuCu_11_D**]: ESI-MS: *m*/*z* 2543.37 Da (calcd. *m*/*z* 2543.35 Da) for [AuCu_11_(D){S_2_P(O*^i^*Pr)_2_}_6_(C≡CPh)_3_ + Cu^+^]^+^. ^1^H NMR (400 MHz, CDCl_3_): 7.35 (8H, d, C_6_*H*_5_), 7.63 (7H, d, C_6_*H*_5_), 4.67–5.05 (12H, m, OC*H*), and 1.05–1.42 (72H, dd, C*H*_3_). ^31^P NMR (121.5 MHz, CDCl_3_): 104.45, 100.50, and 99.91 ppm. ^2^H NMR (400 MHz, CHCl_3_): −1.41 (1D, s, μ_2_-*D*) ppm. 

#### 3.2.2. [AuCu11(H){S2P(OnPr)2}6(C≡CPh)3]

**[AuCu_11_(H){S_2_P(O*^n^*Pr)_2_}_6_(C≡CPh)_3_]**: ESI-MS: *m*/*z* 2542.37 Da (calcd. *m*/*z* 2542.34 Da) for [AuCu_11_(H){S_2_P(O*^n^*Pr)_2_}_6_(C≡CPh)_3_ + Cu^+^]^+^; *m*/*z* 2578.32 Da (cacld. *m*/*z* 2578.30 Da) for [AuCu_11_(Cl){S_2_P(O*^n^*Pr)_2_}_6_(C≡CPh)_3_ + Cu^+^]^+^; *m*/*z* 2642.39 Da (calcd. *m*/*z* 2642.37 Da) for [AuCu_12_{S_2_P(O*^n^*Pr)_2_}_6_(C≡CPh)_4_]^+^. ^1^H NMR (400 MHz, CDCl_3_): 7.48–7.70 (15H, C_6_*H*_5_), 3.94–4.26 (24H, OC*H_2_*), 1.27–1.73 (24H, C*H*_2_), 0.72–1.01 (36H, C*H_3_*), and −1.48 (1H, μ_2_-H) ppm. ^31^P NMR (121.5 MHz, CDCl_3_): 109.7, 106.2, and 105.9 ppm.

**[AuCu_11_(D){S_2_P(O*^n^*Pr)_2_}_6_(C≡CPh)_3_]:** ESI-MS: *m*/*z* 2543.44 Da (calcd. *m*/*z* 2543.35 Da) for [AuCu_11_(D){S_2_P(O*^n^*Pr)_2_}_6_(C≡CPh)_3_ + Cu^+^]^+^; *m*/*z* 2642.46 Da (calcd. *m*/*z* 2642.37 Da) for [AuCu_12_{S_2_P(O*^n^*Pr)_2_}_6_(C≡CPh)_4_]^+^. ^1^H NMR (400 MHz, CDCl_3_): 7.37–7.59 (15H, C_6_*H*_5_), 3.99–4.26 (24H, OC*H_2_*), 1.34–1.76 (24H, C*H*_2_), and 0.75-.0.98 (36H, C*H_3_*). ^31^P NMR (121.5 MHz, CDCl_3_): 110.1 106.3, and 105.4 ppm. ^2^H NMR (400 MHz, CHCl_3_): −1.40 (1D, s, *D*) ppm.

#### 3.2.3. [AuCu_11_(H){S_2_P(O^i^Bu)_2_}_6_(C≡CPhOCH_3_)_3_]

**[AuCu_11_(H){S_2_P(O*^i^*Bu)_2_}_6_(C≡CPhOCH_3_)_3_]**: ESI-MS: *m*/*z* 2800.59 Da (calcd. *m*/*z* 2800.56 Da) for [AuCu_11_(H){S_2_P(O*^i^*Bu)_2_}_6_(C≡CPhOCH_3_)_3_+Cu^+^]^+^; *m*/*z* 2834.52 Da (cacld. *m*/*z* 2834.52 Da) for [AuCu_11_(Cl){S_2_P(O*^i^*Bu)_2_}_6_(C≡CPhOCH_3_)_3_+Cu^+^]^+^; *m*/*z* 2932.64 Da (calcd. *m*/*z* 2932.66 Da) for [AuCu_12_{S_2_P(O*^i^*Bu)_2_}_6_(C≡CPhOCH_3_)_4_]^+^. ^1^H NMR (400 MHz, CDCl_3_): 6.86–7.57 (12H, C_6_*H*_5_OCH_3_), 3.71–4.00 (24H, OC*H*), 3.81 (3H, C_6_H_5_OC*H_3_*), 1.55–1.72 (12H, C*H*_2_), 0.76–1.00 (36H, C*H*_3_), and −1.63 (1H, μ_2_-H) ppm. ^31^P NMR (121.5 MHz, CDCl_3_): 109.6, 105.6, and 104.9 ppm. 

**[AuCu_11_(D){S_2_P(O*^i^*Bu)_2_}_6_(C≡CPhOCH_3_)_3_]:** ESI-MS: *m*/*z* 2801.55 Da (calcd. *m*/*z* 2801.57 Da) for [AuCu_11_(D){S_2_P(O*^i^*Bu)_2_}_6_(C≡CPhOCH_3_)_3_+Cu^+^]^+^; *m*/*z* 2672.52 Da (calcd. *m*/*z* 2671.70 Da) for [AuCu_11_(D){S_2_P(O*^i^*Bu)_2_}_6_(C≡CPhOCH_3_)_3_]. ^1^H NMR (400 MHz, CDCl_3_): 6.86–7.57 (12H, C_6_*H*_5_OCH_3_), 3.71–4.00 (24H, OC*H*), 3.81 (3H, C_6_H_5_OC*H_3_*), 1.55–1.72 (12H, C*H*_2_), and 0.76–1.00 (36H, C*H*_3_). ^31^P NMR (121.5 MHz, CDCl_3_): 109.6, 105.6, and 104.9 ppm. ^2^H NMR (400 MHz, CHCl_3_): −1.74 (1D, s, *D*) ppm.

### 3.3. Computational Details

Geometry optimizations were performed by DFT calculations with the Gaussian 16 package [56] using the PBE0 functional [57] and the all-electron Def2-TZVP basis set from the EMSL Basis Set Exchange Library [58]. All the optimized geometries were characterized as true minima on their potential energy surface by harmonic vibrational analysis. The Wiberg bond indices were computed with the NBO 6.0 program [59]. The UV–visible transitions were calculated by means of TD-DFT calculations [60]. Only singlet–singlet, i.e., spin-allowed, transitions were computed. The UV–visible spectra were simulated from the computed TD-DFT transitions and their oscillator strengths by using the SWizard program [61], each transition being associated with a Gaussian function of half-height width equal to 2000 cm^−1^. The compositions of the molecular orbitals were calculated using the AOMix program [62], and the NMR chemical shifts were calculated by using the gauge-including atomic orbital (GIAO) method [63]. All calculations correspond to the molecule in vacuum.

## 4. Conclusions

We report here the synthesis and characterization of the bimetallic hydride cluster **AuCu_11_H**. The combination of spectroscopic methods and DFT calculations allowed establishing that **AuCu_11_H** has a similar structure as its **AuCu_11_Cl** relative. Its ligand-protected metallic core is composed of a defective Au@Cu_11_ cuboctahedron. **AuCu_11_H** is metastable and, in a chlorinated solvent, undergoes a spontaneous ligand-exchange transformation into **AuCu_11_Cl** and aggregates into the **AuCu_12_** NC of increased nuclearity. 

## Figures and Tables

**Table 1:** The positive-mode ESI-MS of [**AuCu_11_H**+Cu^+^]^+^.

No.	Cluster Code	*m*/*z* Exp.	*m*/*z* Cacl.
1.	[**AuCu_11_H** + Cu^+^]^+^	2542.03	2542.34
2.	[**AuCu_11_Cl** + Cu^+^]^+^	2575.96	2575.31
3.	[**AuCu_12_**]^+^	2642.37	2642.37

**Table 2:** Selected DFT-computed data for **AuCu_11_H** and **AuCu_11_Cl** ^a^.

		AuCu_11_H	AuCu_11_Cl ^a^
HOMO-LUMO gap (eV)		3.51	3.59
Interatomic distances (Å) and Wiberg indices (avg. values)	Au-Cu	2.790 [0.062]	2.814 [0.060]
	Cu-Cu	2.777 [0.040]	2.789 [0.041]
	Cu-H/Cl	1.633 [0.273]	2.272 [0.198]
Natural atomic charges (avg. values)	Au	−0.69	−0.73
	Cu	0.73	0.72
	H/Cl	−0.53	−0.73

^a^ From ref. [25].

## Data Availability

The original contributions presented in the study are included in the article/Supplementary Material; further inquiries can be directed to the corresponding author/s.

## Supplementary Materials

The following supporting information can be downloaded at https://www.mdpi.com/article/10.3390/molecules29184427/s1, Figure S1. Comparison between the experimental data (top) and the simulated (bottom) isotope patterns of peaks (a) [**AuCu_11_Cl**+Cu^+^]^+^ and (b) [**AuCu_12_**]^+^; Figure S2. ESI-MS spectra [**AuCu_11_D**+Cu^+^]^+^. The insets show the comparisons between the experimental data (top) and the simulated (bottom) isotope patterns for [**AuCu_11_D**+Cu^+^]^+^; Figure S3. Comparisons between the experimental data (top) and the simulated (bottom) isotope patterns of peaks (a) [**AuCu_11_D**] and (b) [**AuCu_12_**]^+^; Figure S4. ^1^H NMR spectrum of **AuCu_11_D** in CDCl_3_; Figure S5. ^31^P{^1^H} NMR spectrum of **AuCu_11_H** in CDCl_3_; Figure S6. ^31^P{^1^H} NMR spectrum of **AuCu_11_D** in CDCl_3_; Figure S7. ^13^C NMR spectrum of **AuCu_11_H** in *d_6_*-acetone; Figure S8. The ESI-MS spectrum of [AuCu_11_(H){S_2_P(O*^n^*Pr)_2_}_6_(C≡CPh)_3_+Cu^+^]^+^ (I), [AuCu_11_(Cl){S_2_P(O*^n^*Pr)_2_}_6_(C≡CPh)_3_+Cu^+^]^+^ (II), and [AuCu_12_{S_2_P(O*^n^*Pr)_2_}_6_(C≡CPh)_4_]^+^ (III); Figure S9. Comparisons between the experimental data (top) and the simulated (bottom) isotope patterns of peaks (a) [AuCu_11_(H){S_2_P(O*^n^*Pr)_2_}_6_(C≡CPh)_3_+Cu^+^]^+^ (I), (b) [AuCu_11_(Cl){S_2_P(O*^n^*Pr)_2_}_6_(C≡CPh)_3_+Cu^+^]^+^ (II), and (c) [AuCu_12_{S_2_P(O*^n^*Pr)_2_}_6_(C≡CPh)_4_]^+^ (III); Figure S10. The ESI-MS spectrum of [AuCu_11_(D){S_2_P(O*^n^*Pr)_2_}_6_(C≡CPh)_3_+Cu^+^]^+^ (I) and [AuCu_12_{S_2_P(O*^n^*Pr)_2_}_6_(C≡CPh)_4_]^+^ (II); Figure S11. Comparisons between the experimental data (top) and the simulated (bottom) isotope patterns of peaks (a) [AuCu_11_(D){S_2_P(O*^n^*Pr)_2_}_6_(C≡CPh)_3_+Cu^+^]^+^ (I) and (b) [AuCu_12_{S_2_P(O*^n^*Pr)_2_}_6_(C≡CPh)_4_]^+^ (II); Figure S12. The ESI-MS spectrum of [AuCu_11_(H){S_2_P(O*^i^*Bu)_2_}_6_(C≡CPhOCH_3_)_3_+Cu^+^]^+^ (I), [AuCu_11_(Cl){S_2_P(O*^i^*Bu)_2_}_6_(C≡CPhOCH_3_)_3_+Cu^+^]^+^ (II), and [AuCu_12_{S_2_P(O*^i^*Bu)_2_}_6_(C≡CPhOCH_3_)_4_]^+^ (III); Figure S13. Comparisons between the experimental data (top) and the simulated (bottom) isotope patterns of peaks (a) [AuCu_11_(H){S_2_P(O*^i^*Bu)_2_}_6_(C≡CPhOCH_3_)_3_+Cu^+^]^+^ (I), (b) [AuCu_11_(Cl){S_2_P(O*^i^*Bu)_2_}_6_(C≡CPhOCH_3_)_3_+Cu^+^]^+^ (II), and (c) [AuCu_12_{S_2_P(O*^i^*Bu)_2_}_6_(C≡CPhOCH_3_)_4_]^+^ (II); Figure S14. The ESI-MS spectrum of [AuCu_11_(D){S_2_P(O*^i^*Bu)_2_}_6_(C≡CPhOCH_3_)_3_+Cu^+^]^+^ (I) and [AuCu_10_{S_2_P(O*^i^*Bu)_2_}_6_(C≡CPhOCH_3_)_3_]^+^ (II). Inset: Comparisons between the experimental data (top) and the simulated (bottom) isotope pattern of peak (a) [AuCu_11_(D){S_2_P(O*^i^*Bu)_2_}_6_(C≡CPhOCH_3_)_3_+Cu^+^]^+^ (I); Figure S15. ^1^H NMR spectrum of [AuCu_11_(H){S_2_P(O*^n^*Pr)_2_}_6_(C≡CPh)_3_] in CDCl_3_; Figure S16. ^31^P{^1^H} NMR spectrum of [AuCu_11_(H){S_2_P(O*^n^*Pr)_2_}_6_(C≡CPh)_3_] in CDCl_3_; Figure S17. ^2^H NMR spectrum of [AuCu_11_(D){S_2_P(O*^n^*Pr)_2_}_6_(C≡CPh)_3_] in CHCl_3_; Figure S18. ^1^H NMR spectrum of [AuCu_11_(H){S_2_P(O*^i^*Bu)_2_}_6_(C≡CPhOCH_3_)_3_] in CDCl_3_; Figure S19. ^31^P{^1^H} NMR spectrum of [AuCu_11_(H){S_2_P(O*^i^*Bu)_2_}_6_(C≡CPhOCH_3_)_3_] in CDCl_3_; Figure S20. ^2^H NMR spectrum of [AuCu_11_(D){S_2_P(O*^i^*Bu)_2_}_6_(C≡CPhOCH_3_)_3_] in CHCl_3_; Figure S21. Comparison of the UV–vis absorption spectra of **AuCu_11_H**, **AuCu_11_Cl**, and **AuCu_12_** in 2-MeTHF solution; Figure S22. The TD-DFT-simulated UV–vis spectrum of **AuCu_11_H**, with individual transitions as vertical bars of lengths proportional to their oscillator strengths; Figure S23. (a) The UV–vis absorption spectrum of **AuCu_11_H**. (b) The excitation spectrum of **AuCu_11_H** in 2-MeTHF solution at ambient temperature; Figure S24. The emission lifetime of cluster **AuCu_11_H** at ambient temperature; Figure S25. Structural transformation of **AuCu_11_H** into **AuCu_12_** in d_6_-acetone monitored by time-dependent ^1^H NMR spectroscopy; Figure S26. ^1^H NMR spectrum of **AuCu_12_** in d_6_-acetone; Figure S27. Structural transformation of **AuCu_11_H** into **AuCu_12_** in d_6_-acetone monitored by time-dependent ^31^P{^1^H} NMR spectroscopy; Figure S28. ^31^P{^1^H} NMR spectrum of **AuCu_12_** in *d_6_*-acetone; Figure S29. Positive-mode ESI-MS spectra of structural transformation of **AuCu_11_H** in acetone; Figure S30. Isotope pattern of (a) **AuCu_11_H** and (b) **AuCu_12_** in structural transformation; inset shows experimental (black) and simulated (red) isotopic distribution pattern; Figure S31. Structural transformation of **AuCu_11_H** into **AuCu_12_** via **AuCu_11_Cl** in CDCl_3_ monitored by time-dependent ^1^H NMR spectroscopy; Figure S32. Structural transformation of **AuCu_11_H** into **AuCu_12_** via **AuCu_11_Cl** in CDCl_3_ monitored by time-dependent ^31^P{^1^H} NMR spectroscopy; Figure S33. ^31^P{^1^H} NMR spectrum of **AuCu_11_Cl** in CDCl_3_; Figure S34. Positive-mode ESI-MS spectra of structural transformation of **AuCu_11_H** in CH_2_Cl_2_; Figure S35. Isotope patten of (a) **AuCu_11_H**, (b) **AuCu_11_Cl**, and (c) **AuCu_12_** in structural transformation; inset shows experimental (black) and simulated (red) isotopic distribution pattern; Figure S36. ^1^H NMR spectrum of **AuCu_11_Cl** in *d_6_*-acetone; Table S1. The 298 K absorption, emission, and lifetime of **AuCu_11_H**, **AuCu_11_Cl**, and **AuCu_12_**; Scheme S1. A proposed mechanism of chloride–hydride ligand exchange, and aggregation within the transformation core of **AuCu_11_H** to **AuCu_12_** via **AuCu_11_Cl** in chloride-containing solvent; Table S2. Cartesian coordinates of the DFT-optimized structure of **AuCu_11_H** and its high-energy isomer.
