# Supplementary material for: Structural Transformation of Metastable Two-Electron Superatom Au-Doped Cu-Rich Alloy Nanocluster
Source: Molecules. 2024 Sep 18;29(18):4427. doi: 10.3390/molecules29184427 (PMC11433815; doi:10.3390/molecules29184427)
Supplement: Supplementary file 1 [file molecules-29-04427-s001.zip › molecules-3159410-supplementary.pdf]

**Electronic Supplementary Materials for**

# Structural Transformation of Metastable Two-Electron Superatom Au-Doped Cu-Rich Alloy Nanocluster

**Rhone P. Brocha Silalahi 1 , Samia Kahlal 2, Jean-Yves Saillard 2,* and C. W. Liu 1,***

[1] Department of Chemistry, National Dong Hwa University, Hualien 97401, Taiwan; rhone.p.brocha.silalahi@gmail.com

[2] Univ Rennes, CNRS, Institut des Sciences Chimiques de Rennes-UMR 6226, F-35000 Rennes, France; kahlal@univ-rennes1.fr

* Correspondence: jean-yves.saillard@univ-rennes1.fr (J.-Y.S.); chenwei@gms.ndhu.edu.tw (C.W.L.); Tel.: +886-3-890-3607 (C.W.L.)

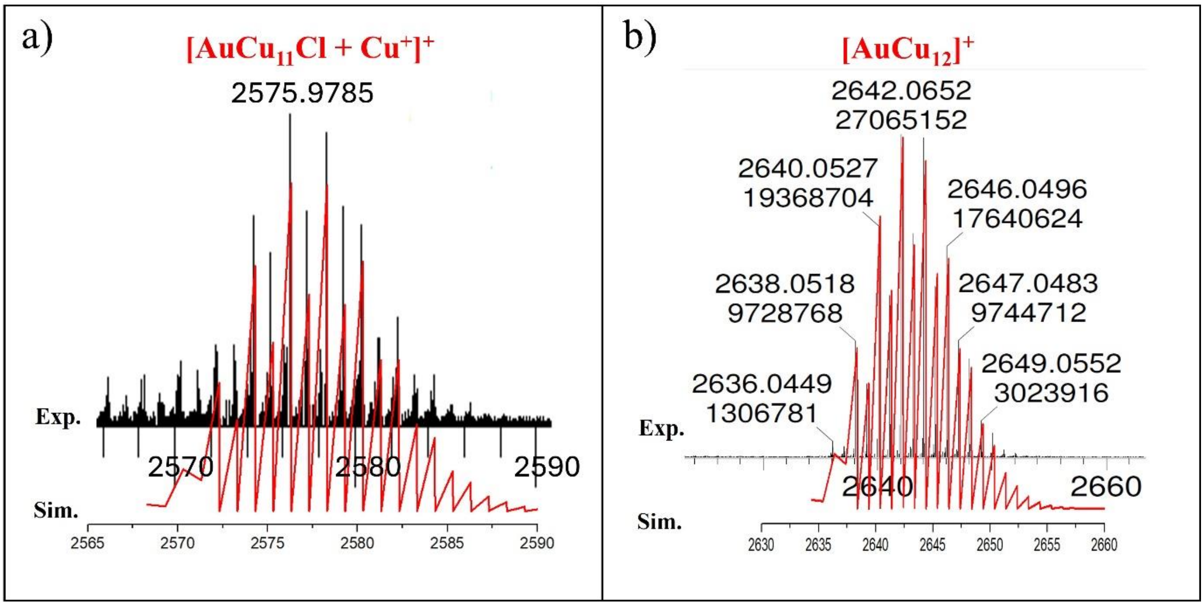


**Figure S1**. Comparison between the experimental data (top) and the simulated (bottom) isotope patterns of peaks (a) [$\mathbf{AuCu_{11}Cl}$ + $Cu^+$]$^+$ and (b) [$\mathbf{AuCu_{12}}$]$^+$.

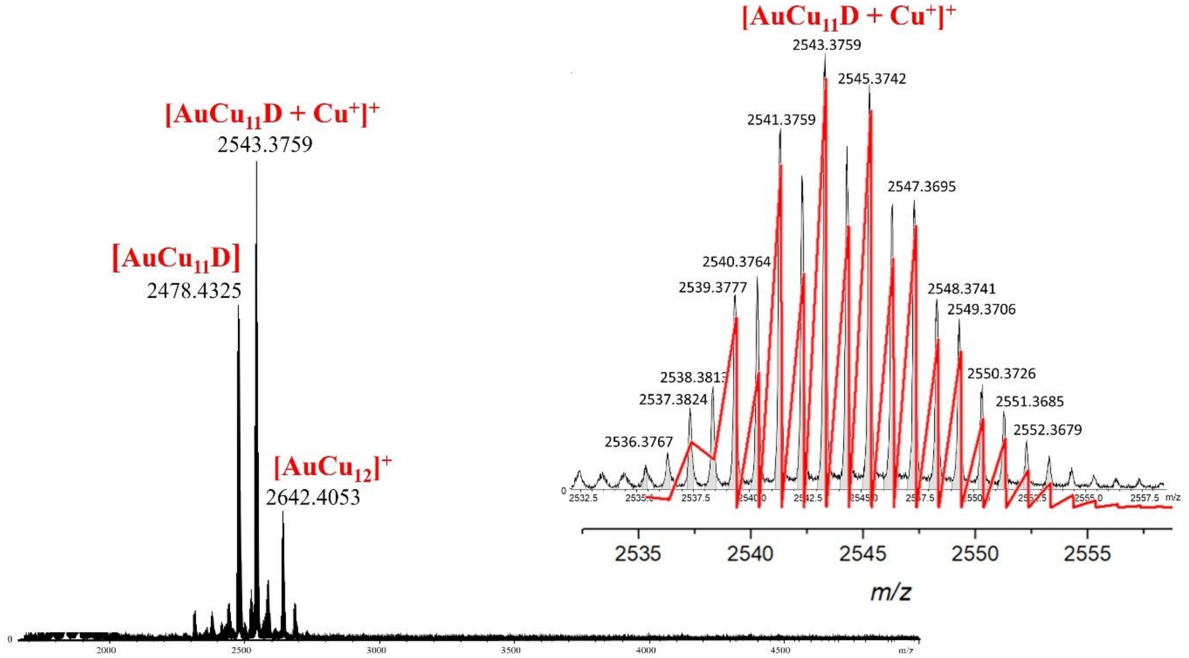


**Figure S2**. ESI-MS spectra [**AuCu₁₁D** + Cu$^{+}$]$^{+}$. The insets show the comparisons between the experimental data (top) and the simulated (bottom) isotope patterns for [**AuCu₁₁D** + Cu$^{+}$]$^{+}$.

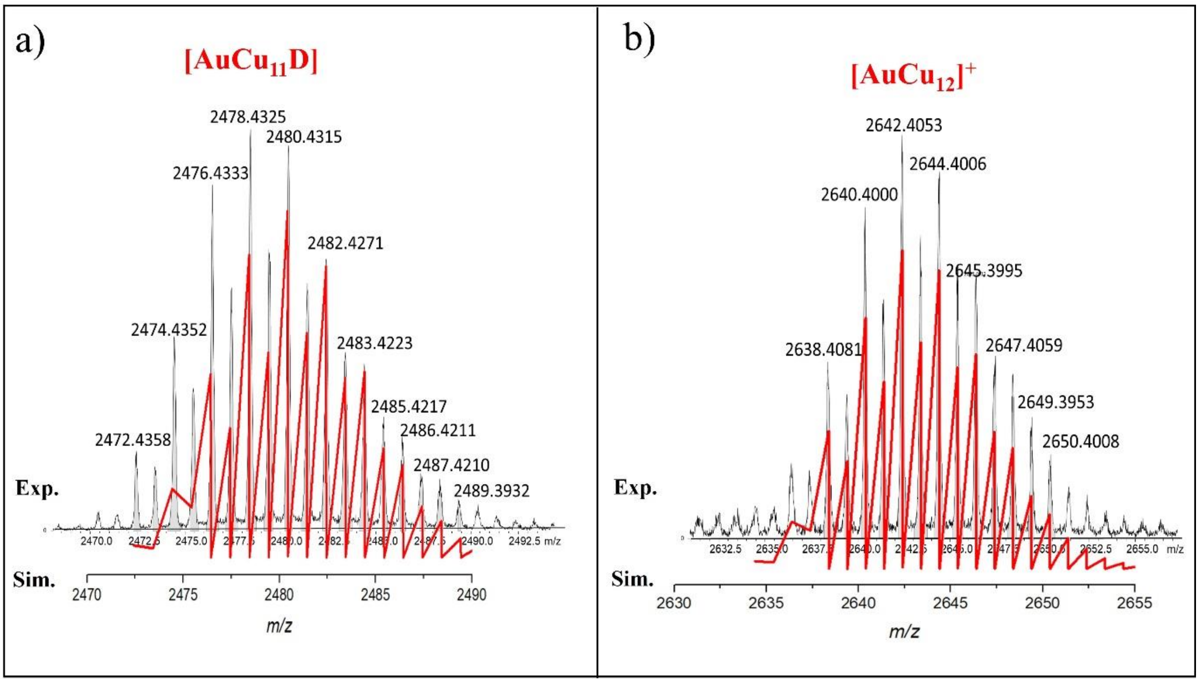


**Figure S3**. Comparisons between the experimental data (top) and the simulated (bottom) isotope patterns of peaks (a) [**$AuCu_{11}D$**] and (b) [**$AuCu_{12}$**]$^{+}$.

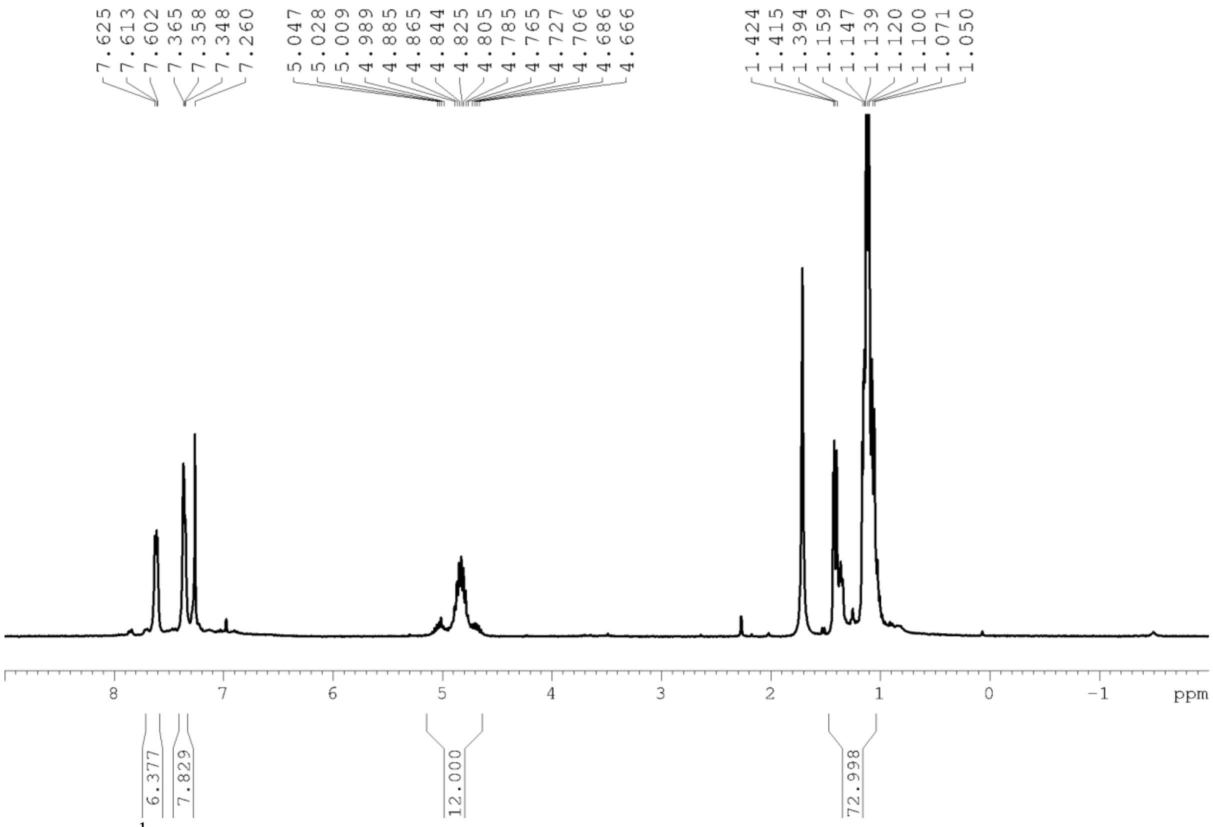


**Figure S4.** $^{1}$H NMR spectrum of **$AuCu_{11}D$** in $CDCl_3$.

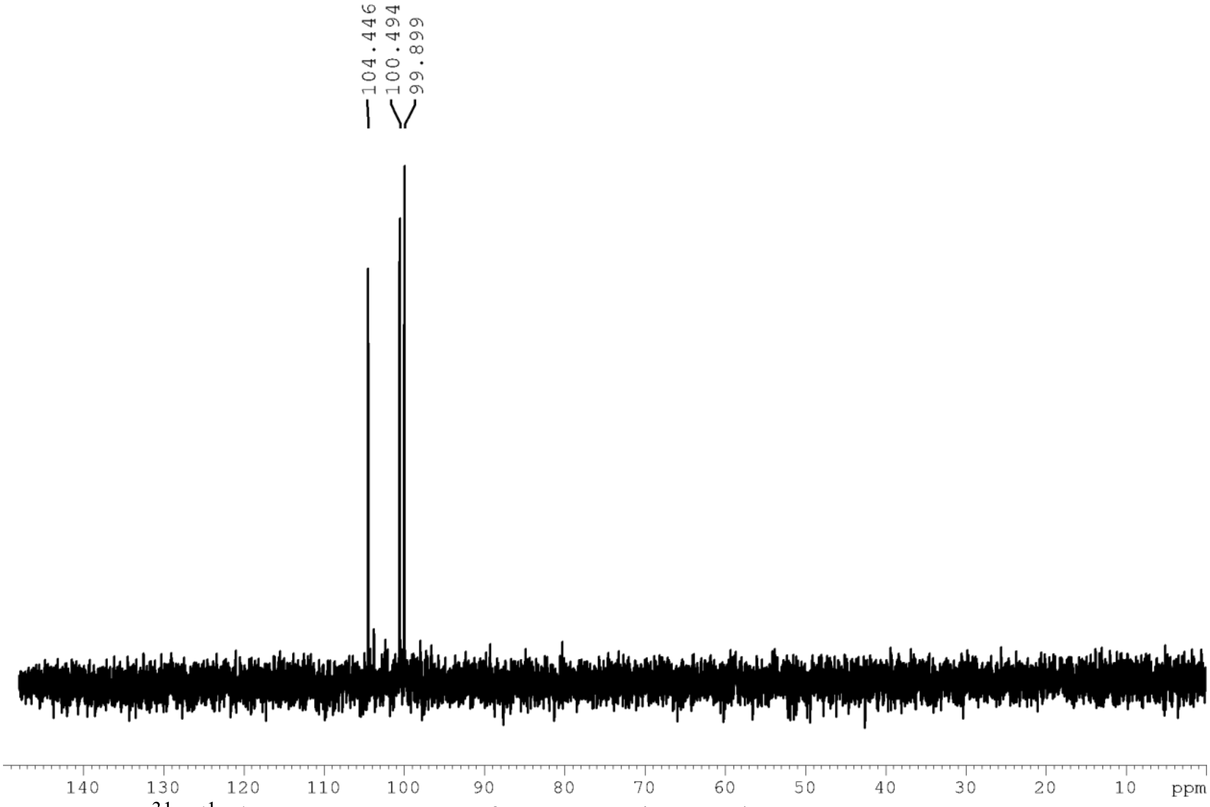

**Figure S5**. $^{31}P\{^{1}H\}$ NMR spectrum of **AuCu$_{11}$H** in $CDCl_3$.

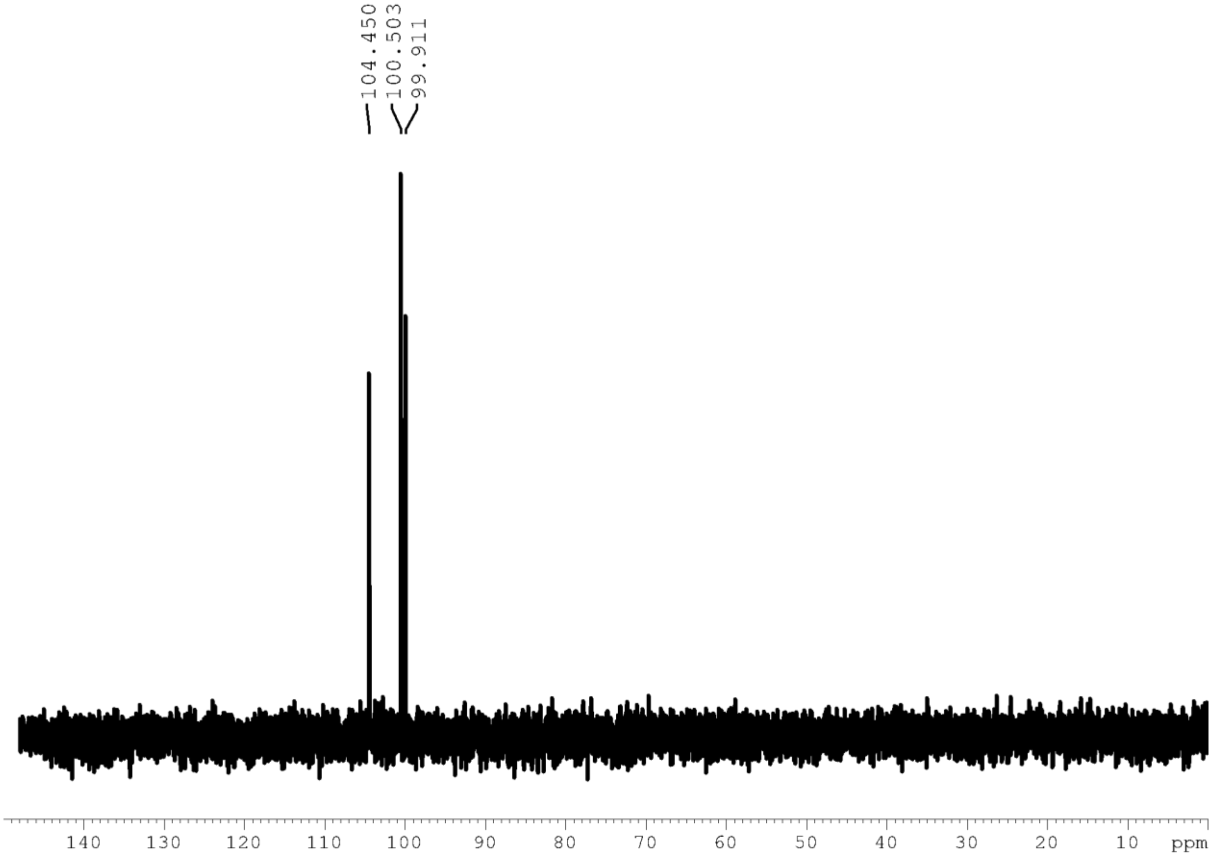


**Figure S6**. $^{31}P\{^{1}H\}$ NMR spectrum of **AuCu$_{11}$D** in $CDCl_3$.

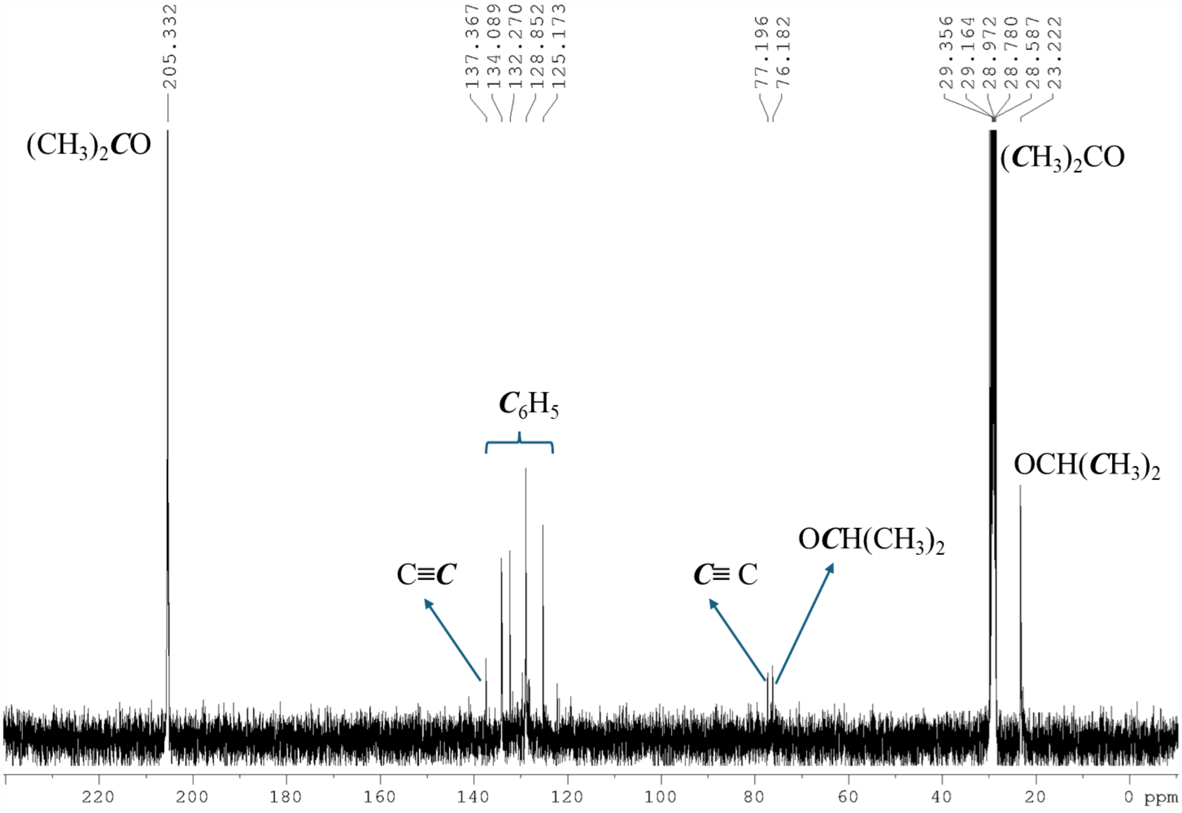


**Figure S7**. $^{13}$C NMR spectrum of **AuCu₁₁H** in *d₆*-acetone.

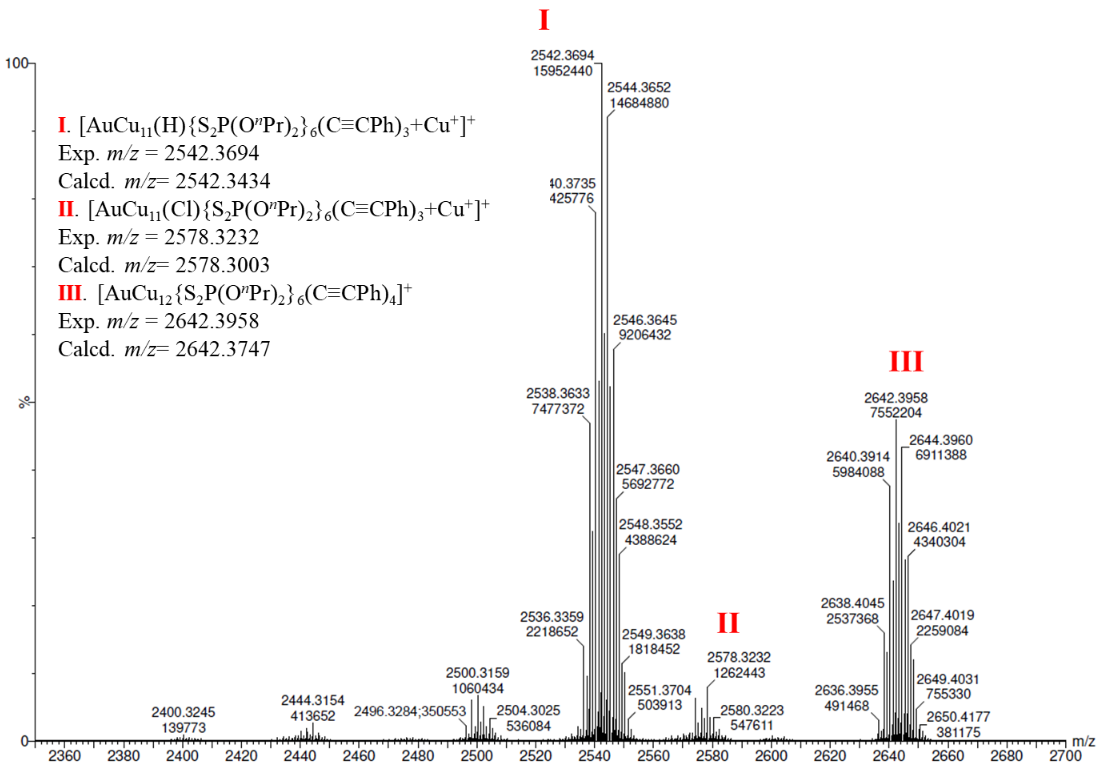


**Figure S8**. The ESI-MS spectrum of $[AuCu_{11}(H)\{S_2P(O^nPr)_2\}_6(C{\equiv}CPh)_3+Cu^+]^+$ (I), $[AuCu_{11}(Cl)\{S_2P(O^nPr)_2\}_6(C{\equiv}CPh)_3+Cu^+]^+$ (II), and $[AuCu_{12}\{S_2P(O^nPr)_2\}_6(C{\equiv}CPh)_4]^+$ (III).

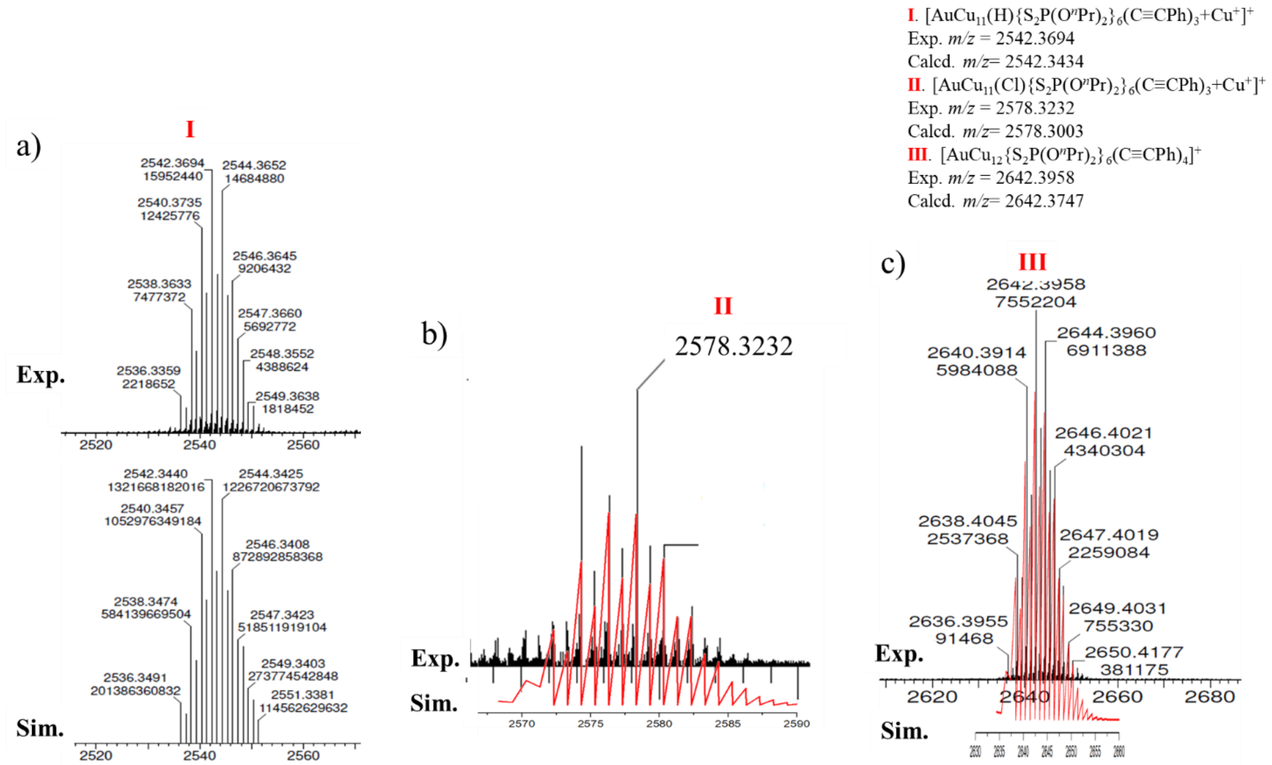


**Figure S9**. Comparisons between the experimental data (top) and the simulated (bottom) isotope patterns of peaks (a) $[AuCu_{11}(H)\{S_2P(O^nPr)_2\}_6(C{\equiv}CPh)_3+Cu^+]^+$ (I), (b) $[AuCu_{11}(Cl)\{S_2P(O^nPr)_2\}_6(C{\equiv}CPh)_3+Cu^+]^+$ (II), and (c) $[AuCu_{12}\{S_2P(O^nPr)_2\}_6(C{\equiv}CPh)_4]^+$ (III).

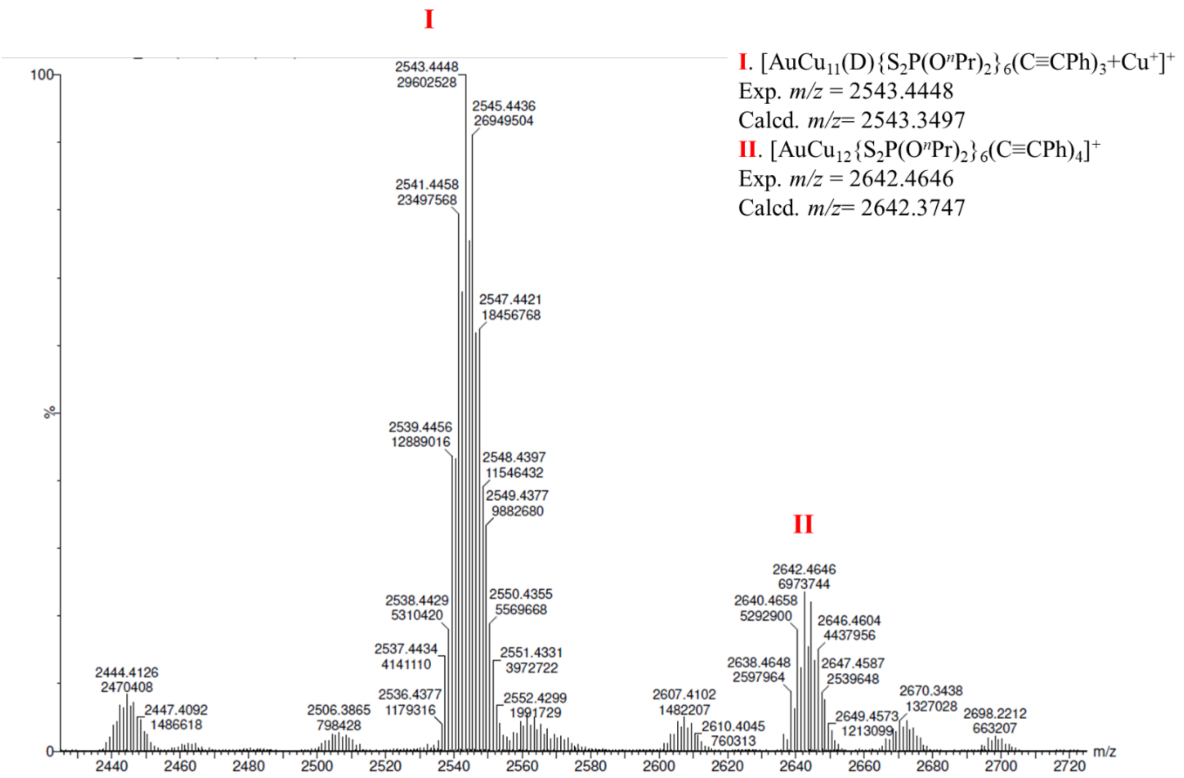


**Figure S10**. The ESI-MS spectrum of $[AuCu_{11}(D)\{S_2P(O^nPr)_2\}_6(C{\equiv}CPh)_3{+}Cu^+]^+$ (I), and $[AuCu_{12}\{S_2P(O^nPr)_2\}_6(C{\equiv}CPh)_4]^+$ (II).

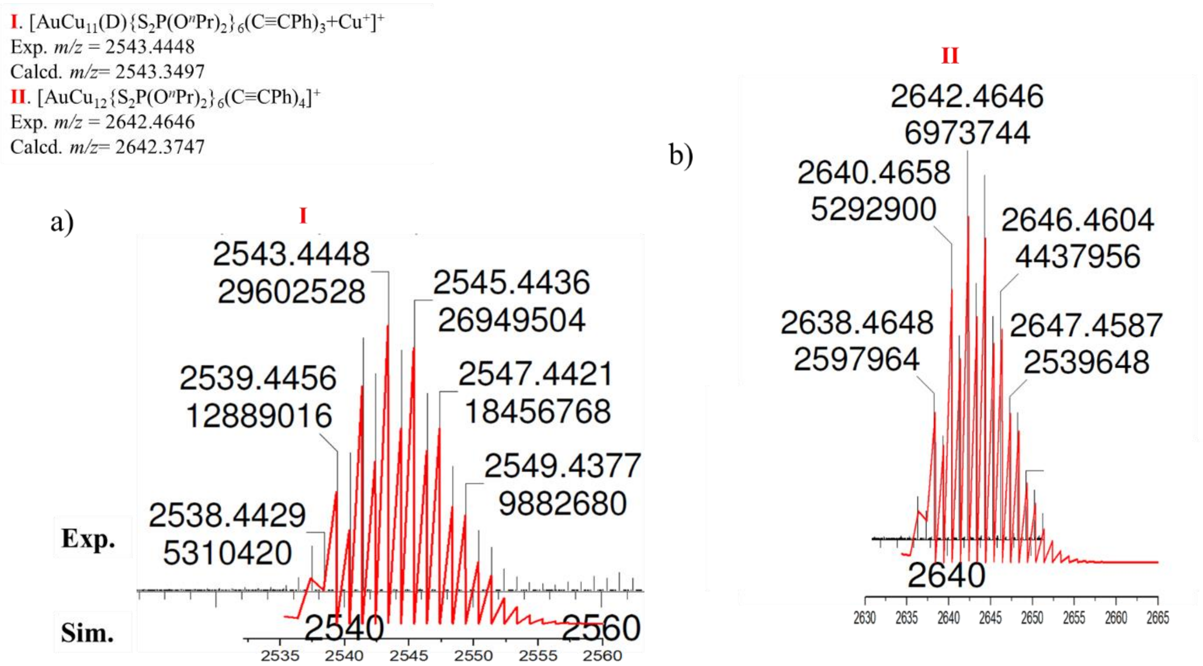


**Figure S11**. Comparisons between the experimental data (top) and the simulated (bottom) isotope patterns of peaks (a) $[AuCu_{11}(D)\{S_2P(O^nPr)_2\}_6(C{\equiv}CPh)_3+Cu^+]^+$ (I), and (b) $[AuCu_{12}\{S_2P(O^nPr)_2\}_6(C{\equiv}CPh)_4]^+$ (II).

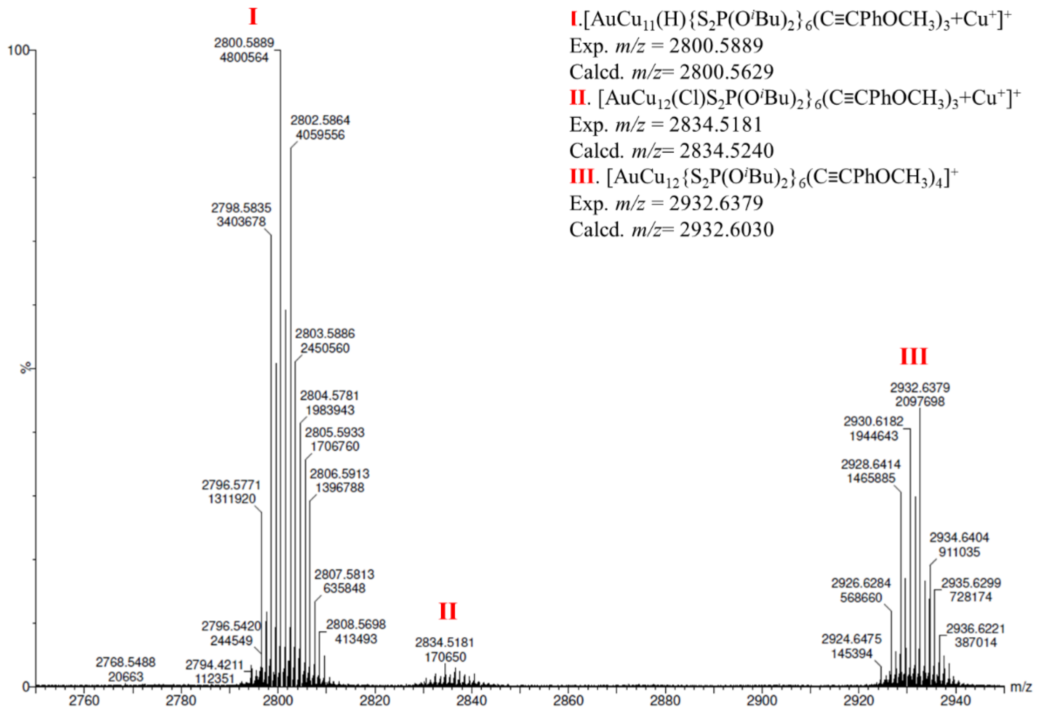


**Figure S12**. The ESI-MS spectrum of $[AuCu_{11}(H)\{S_2P(O^iBu)_2\}_6(C{\equiv}CPhOCH_3)_3{+}Cu^+]^+$ (I), $[AuCu_{11}(Cl)\{S_2P(O^iBu)_2\}_6(C{\equiv}CPhOCH_3)_3{+}Cu^+]^+$ (II), and $[AuCu_{12}\{S_2P(O^iBu)_2\}_6(C{\equiv}CPhOCH_3)_4]^+$ (III).

**I**.$[AuCu_{11}(H)\{S_2P(O^iBu)_2\}_6(C{\equiv}CPhOCH_3)_3+Cu^+]^+$
Exp. *m/z* = 2800.5889
Calcd. *m/z*= 2800.5629
**II**. $[AuCu_{12}(Cl)S_2P(O^iBu)_2\}_6(C{\equiv}CPhOCH_3)_3+Cu^+]^+$
Exp. *m/z* = 2834.5181
Calcd. *m/z*= 2834.5240
**III**. $[AuCu_{12}\{S_2P(O^iBu)_2\}_6(C{\equiv}CPhOCH_3)_4]^+$
Exp. *m/z* = 2932.6379
Calcd. *m/z*= 2932.6030

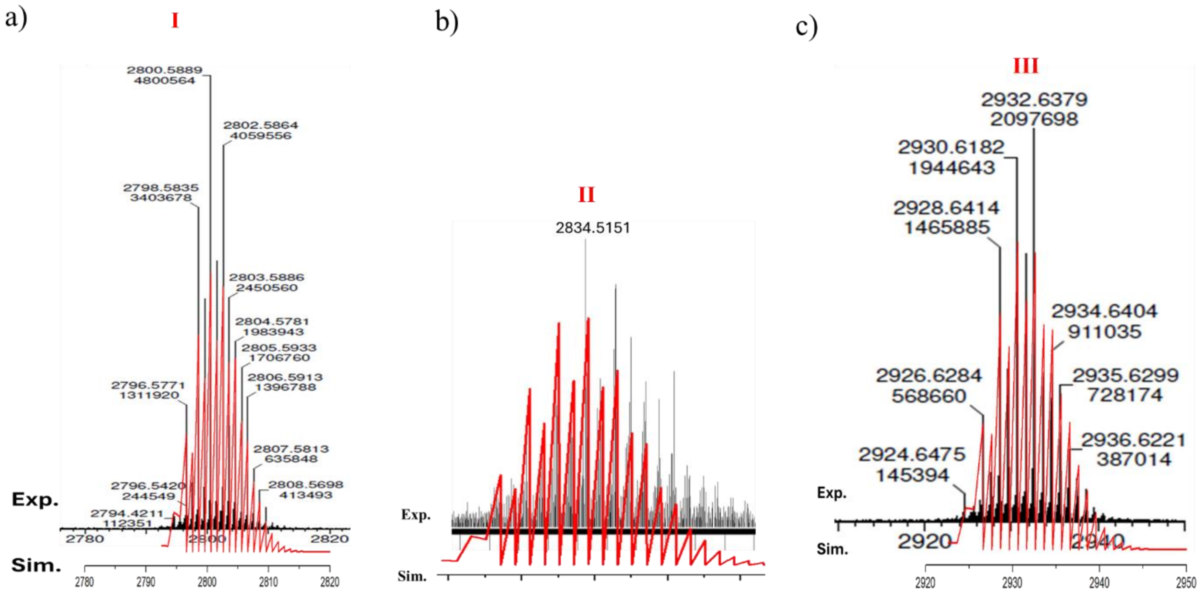


**Figure S13**. Comparisons between the experimental data (top) and the simulated (bottom) isotope patterns of peaks (a) $[AuCu_{11}(H)\{S_2P(O^iBu)_2\}_6(C{\equiv}CPhOCH_3)_3+Cu^+]^+$ (I), (b) $[AuCu_{11}(Cl)\{S_2P(O^iBu)_2\}_6(C{\equiv}CPhOCH_3)_3+Cu^+]^+$ (II), and (c) $[AuCu_{12}\{S_2P(O^iBu)_2\}_6(C{\equiv}CPhOCH_3)_4]^+$ (II).

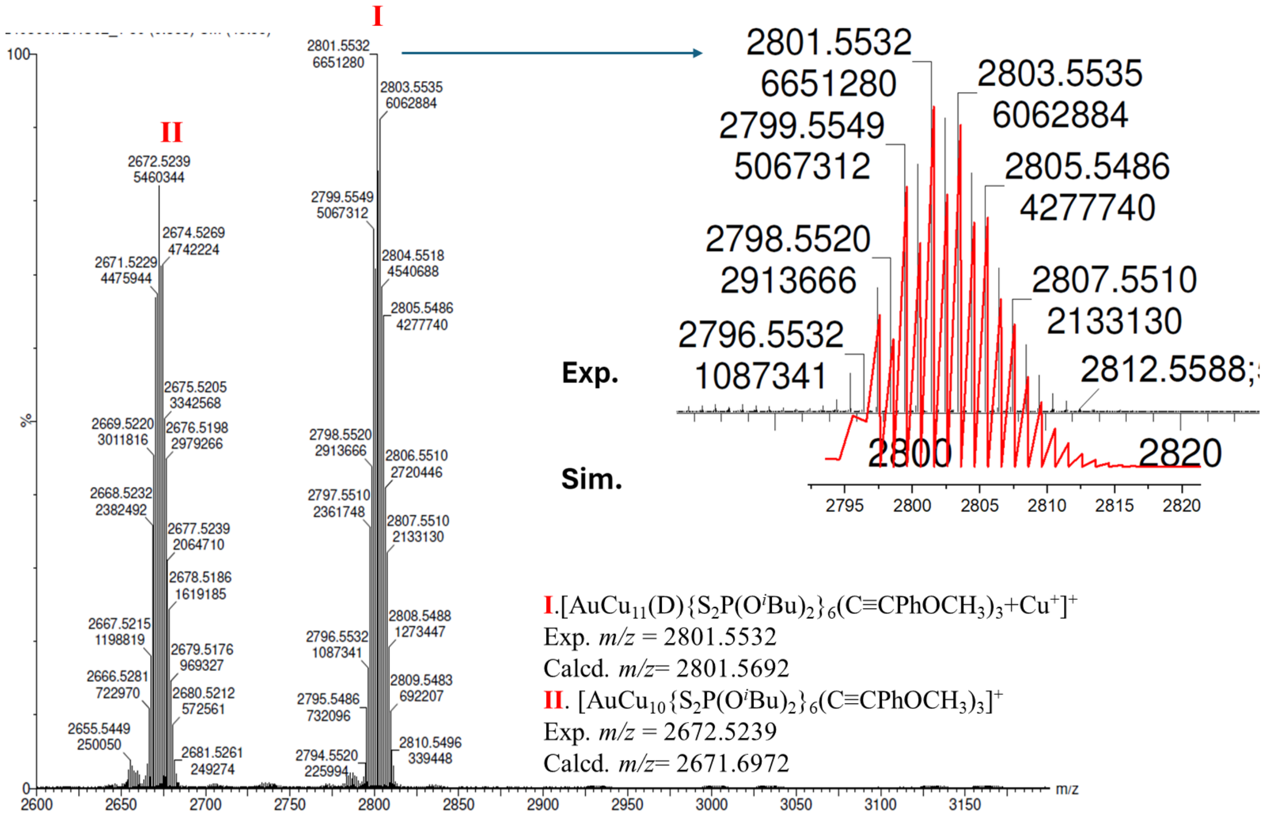


**Figure S14**. The ESI-MS spectrum of $[AuCu_{11}(D)\{S_2P(O^iBu)_2\}_6(C{\equiv}CPhOCH_3)_3{+}Cu^+]^+$ (I), and $[AuCu_{10}\{S_2P(O^iBu)_2\}_6(C{\equiv}CPhOCH_3)_3]^+$ (II). Inset: Comparisons between the experimental data (top) and the simulated (bottom) isotope pattern of peaks $[AuCu_{11}(D)\{S_2P(O^iBu)_2\}_6(C{\equiv}CPhOCH_3)_3{+}Cu^+]^+$ (I).

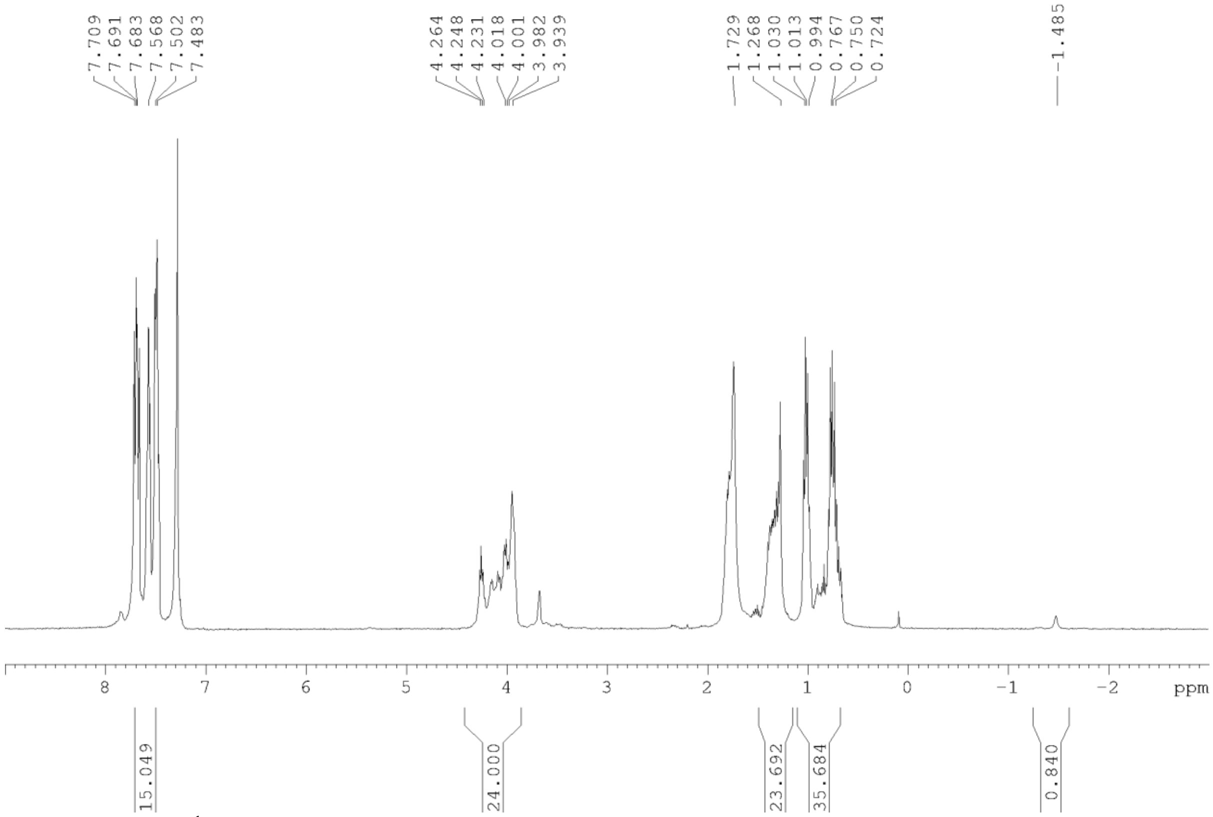


**Figure S15.** $^{1}$H NMR spectrum of $[AuCu_{11}(H)\{S_2P(O^nPr)_2\}_6(C{\equiv}CPh)_3]$ in $CDCl_3$.

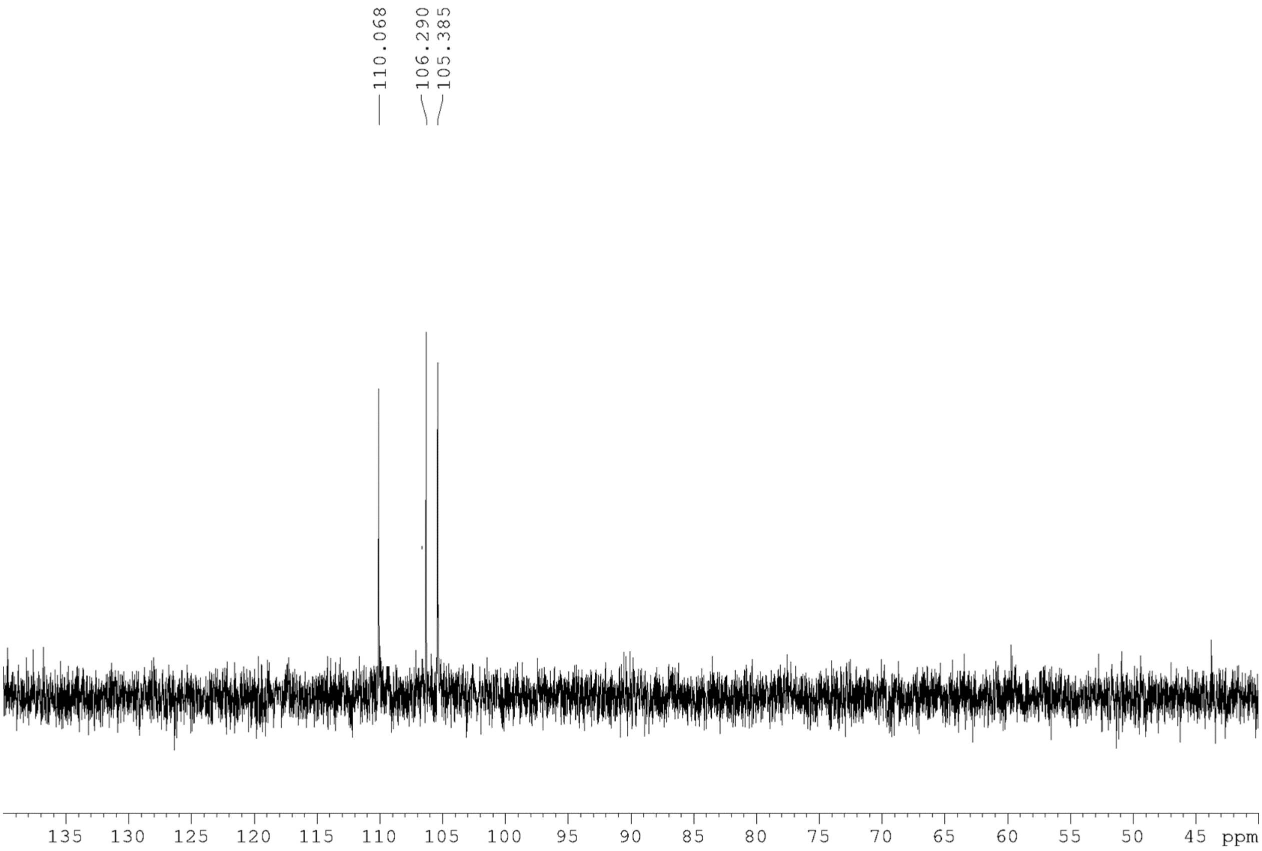


**Figure S16.** $^{31}P\{^1H\}$ NMR spectrum of $[AuCu_{11}(H)\{S_2P(O^nPr)_2\}_6(C{\equiv}CPh)_3]$ in $CDCl_3$.

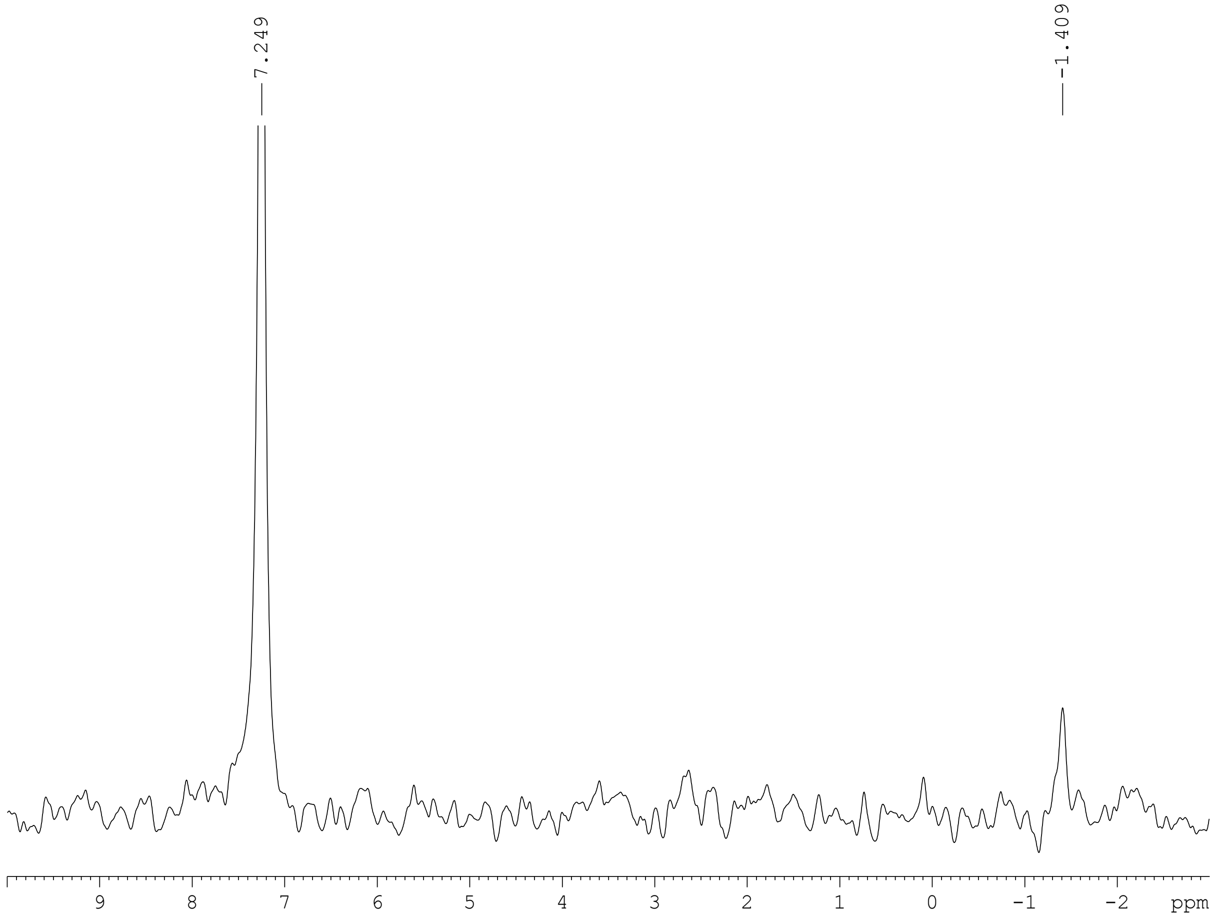


**Figure S17.** $^{2}$H NMR spectrum of [$AuCu_{11}(D)\{S_2P(O^nPr)_2\}_6(C{\equiv}CPh)_3$] in $CHCl_3$.

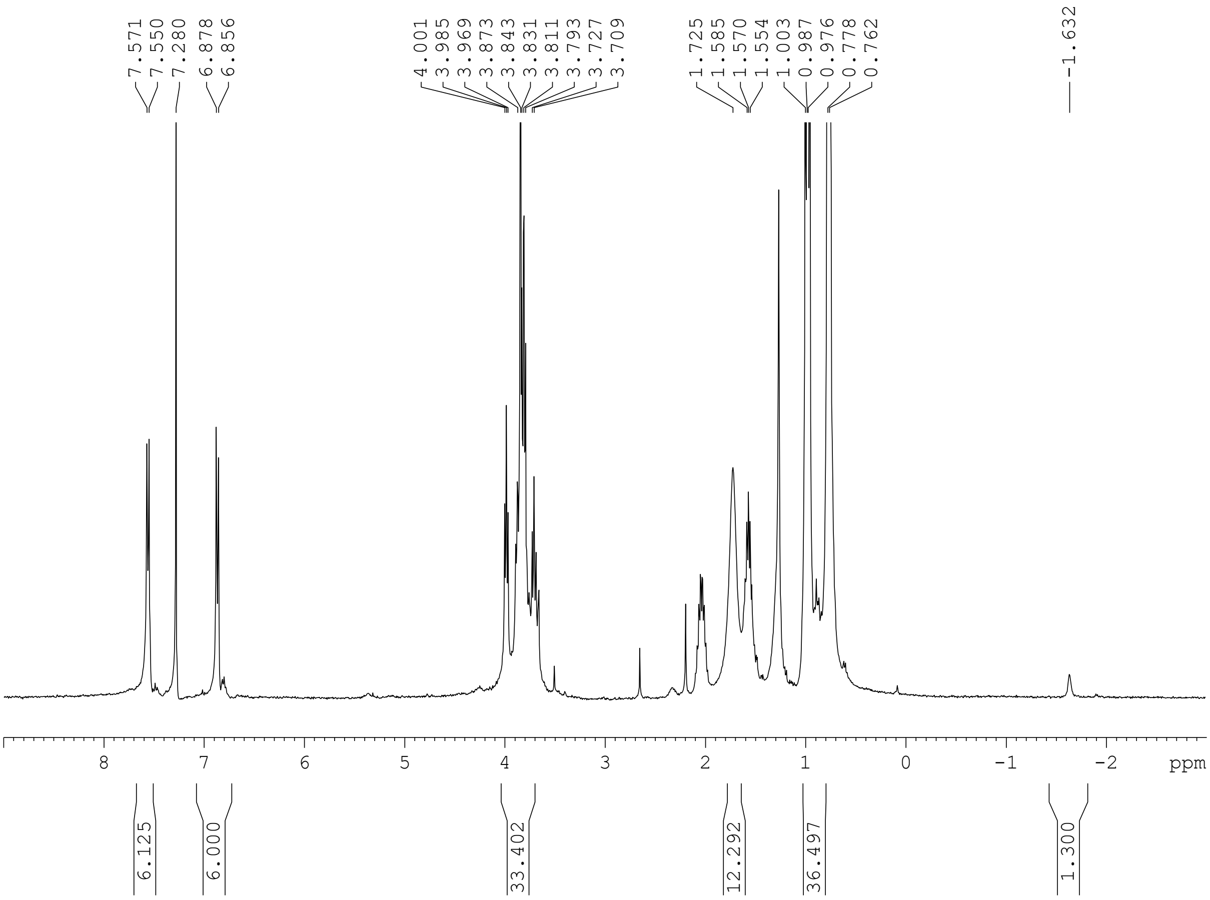


**Figure S18.** $^{1}$H NMR spectrum of $[AuCu_{11}(H)\{S_2P(O^{i}Bu)_2\}_6(C{\equiv}CPhOCH_3)_3]$ in $CDCl_3$.

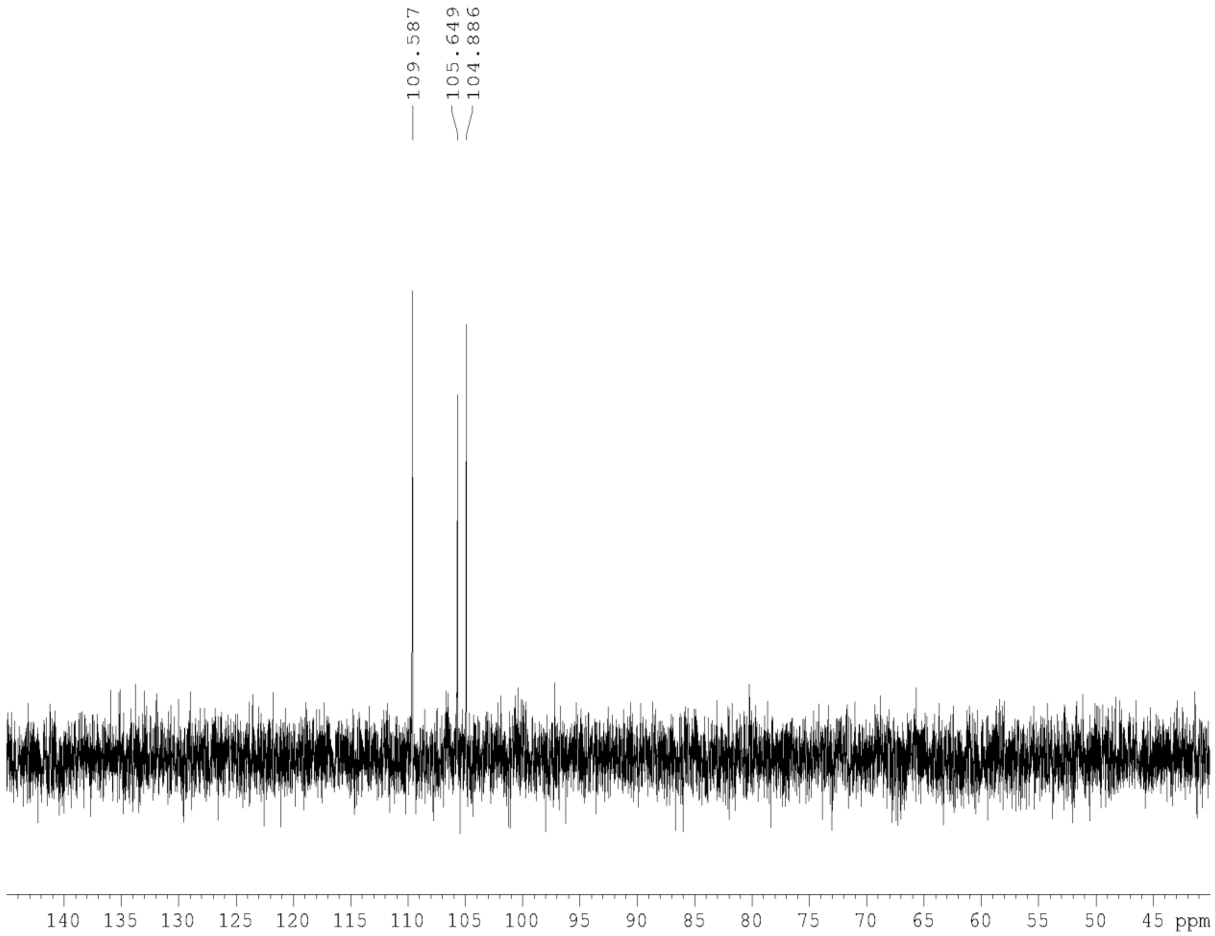


**Figure S19.** $^{31}P\{^{1}H\}$ NMR spectrum of $[AuCu_{11}(H)\{S_2P(O^iBu)_2\}_6(C{\equiv}CPhOCH_3)_3]$ in $CDCl_3$.

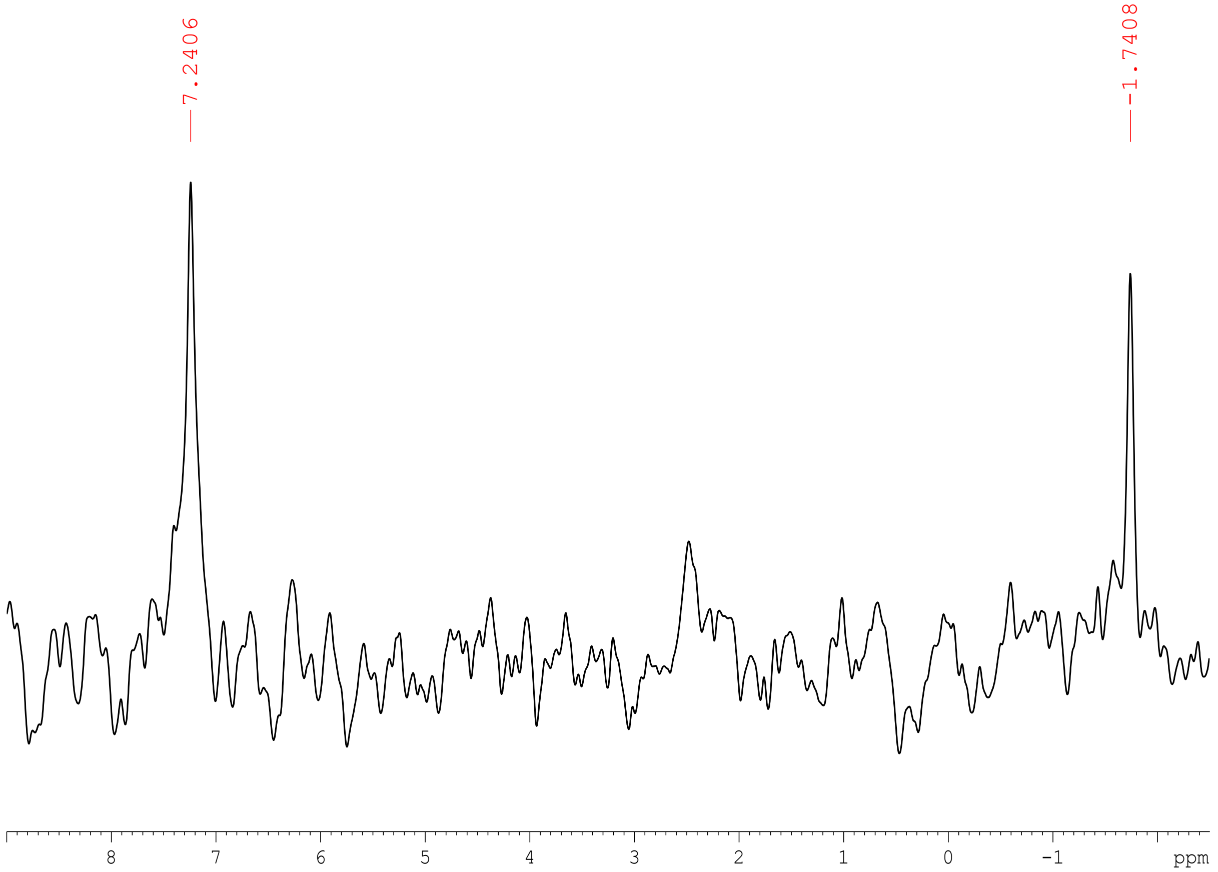


**Figure S20.** $^2$H NMR spectrum of [AuCu$_{11}$(D){S$_2$P(O$^i$Bu)$_2$}$_6$(C≡CPhOCH$_3$)$_3$] in CHCl$_3$.

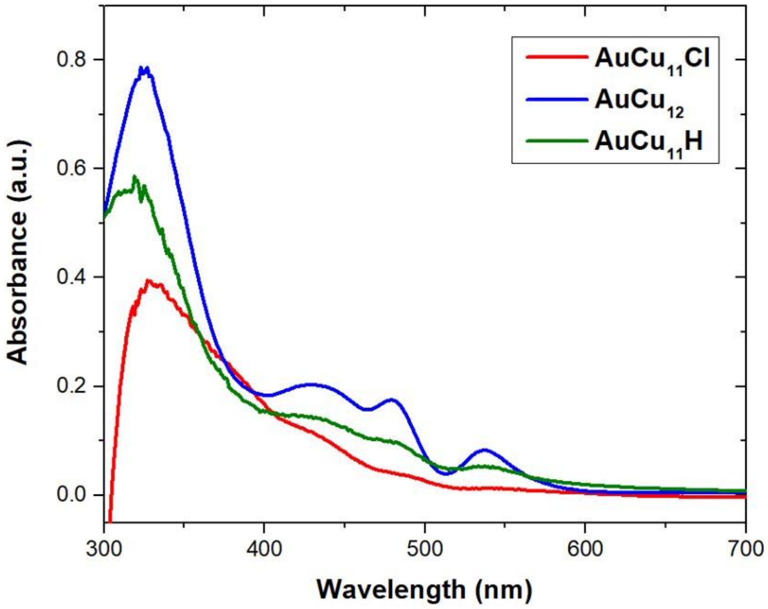


**Figure S21**. Comparison of the UV-vis absorption spectra of **$AuCu_{11}H$**, **$AuCu_{11}Cl$** and **$AuCu_{12}$** in 2-MeTHF solution.

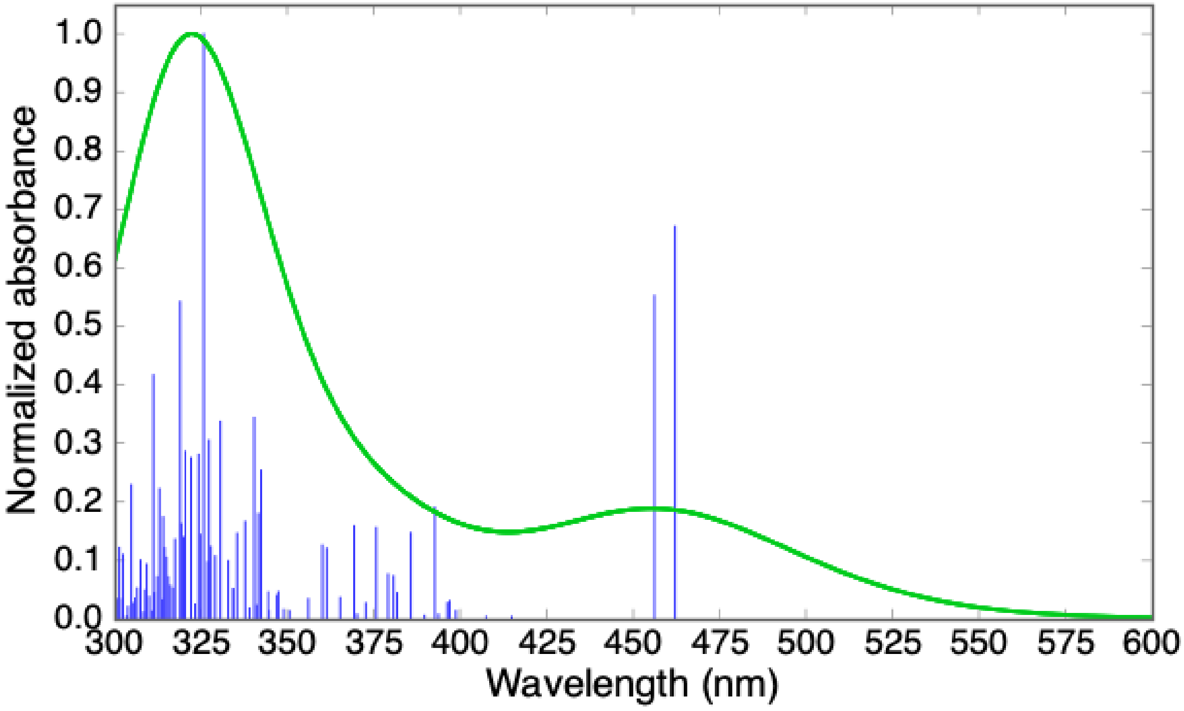


**Figure S22**. The TD-DFT-simulated UV-vis spectrum of **$AuCu_{11}H$**, with individual transitions as vertical bars of lengths proportional to their oscillator strengths.

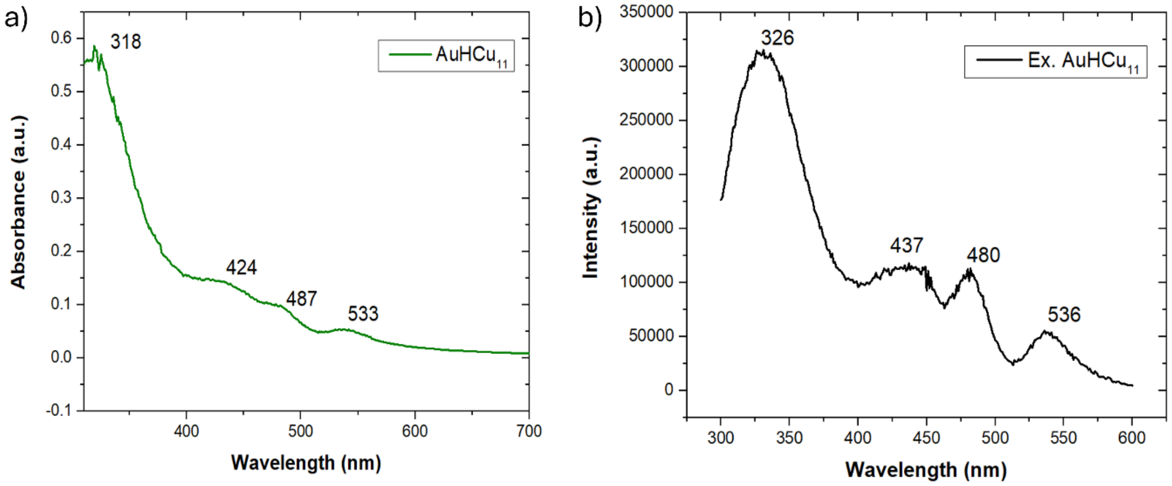


**Figure S23**. (a) The UV-vis absorption spectrum of **$AuCu_{11}H$**. (b) The excitation spectrum of **$AuCu_{11}H$** in 2-MeTHF solution at ambient temperature.

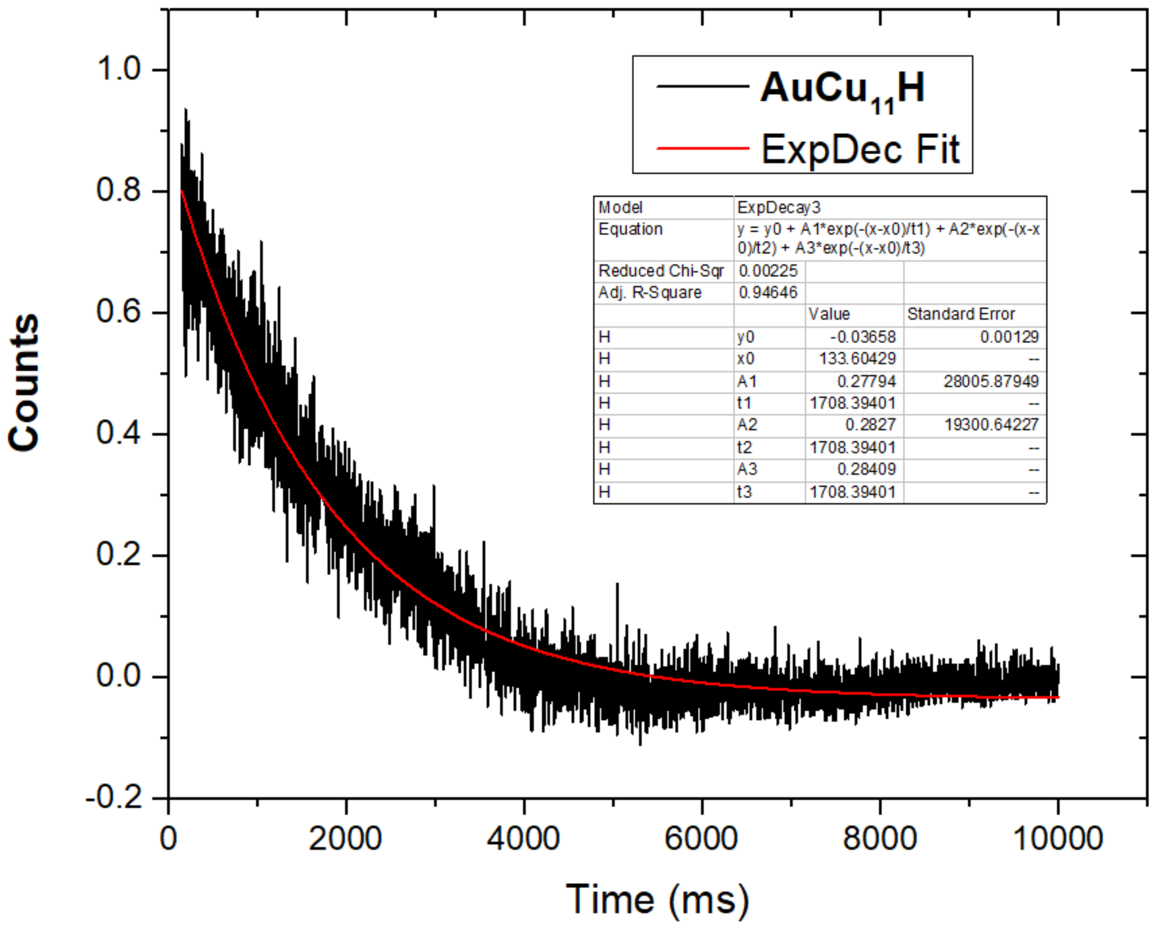


**Figure S24**. The Emission lifetime of cluster **$AuCu_{11}H$** at ambient temperature.

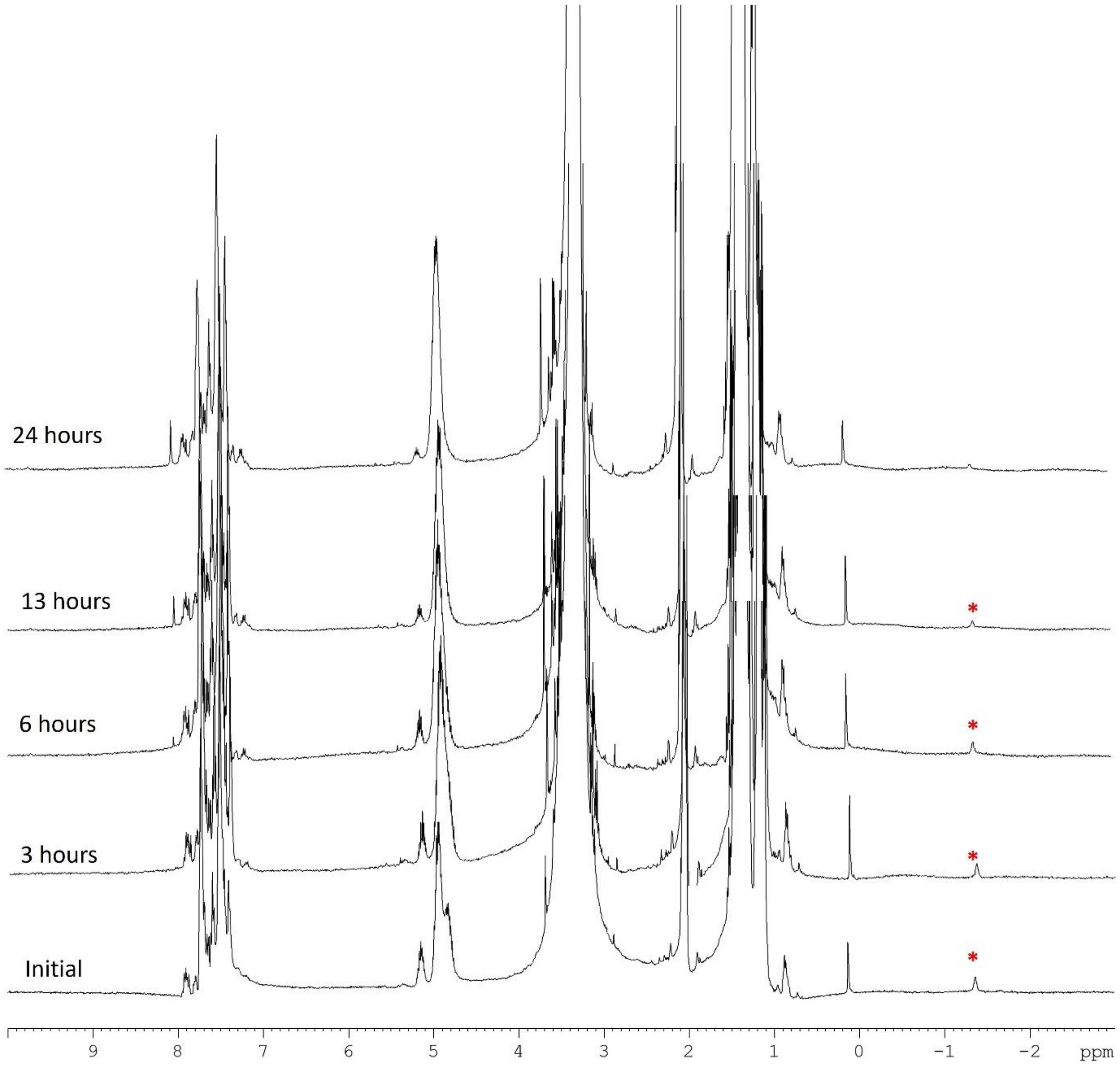


**Figure S25**. Structural transformation of **$AuCu_{11}H$** into **$AuCu_{12}$** in $d_6$-acetone monitored by time-dependent $^1H$ NMR spectroscopy.

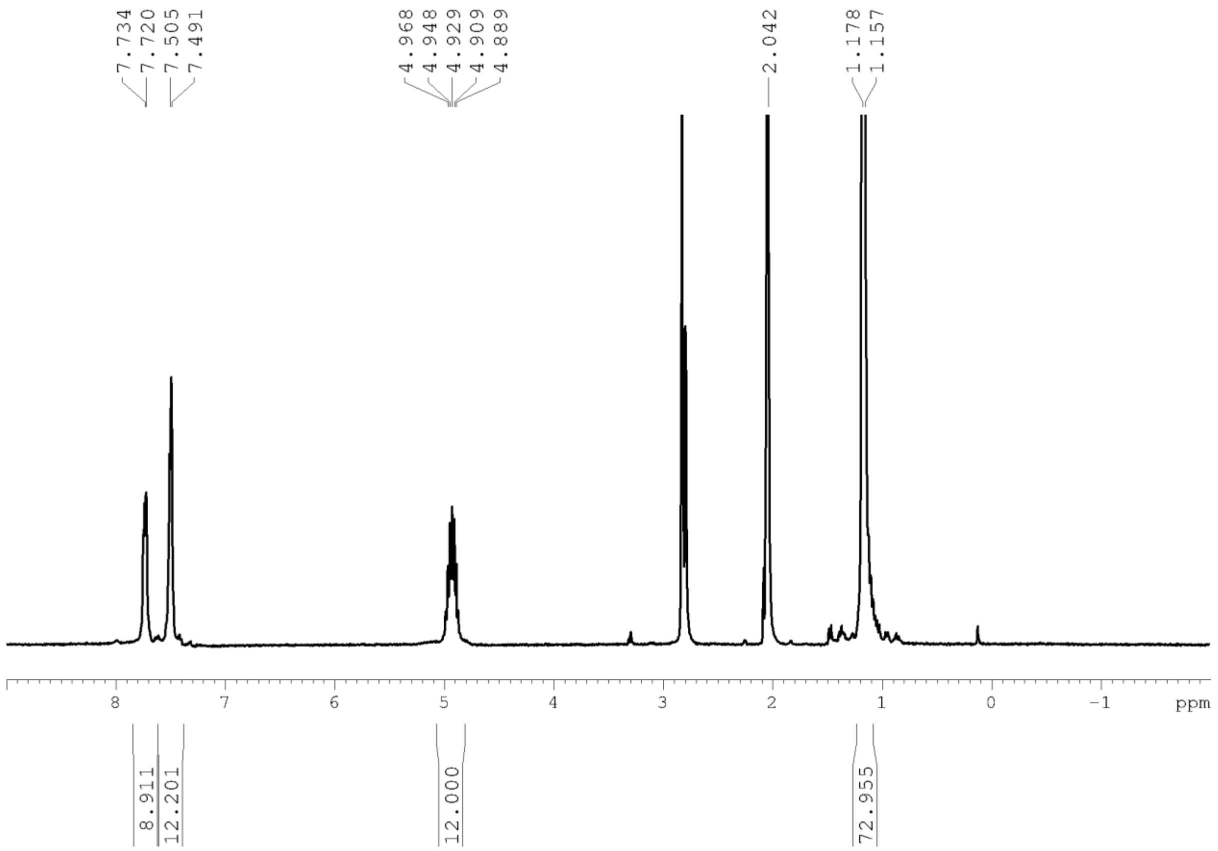


**Figure S26**. $^{1}$H NMR spectrum of **AuCu12** in *d6*-acetone.

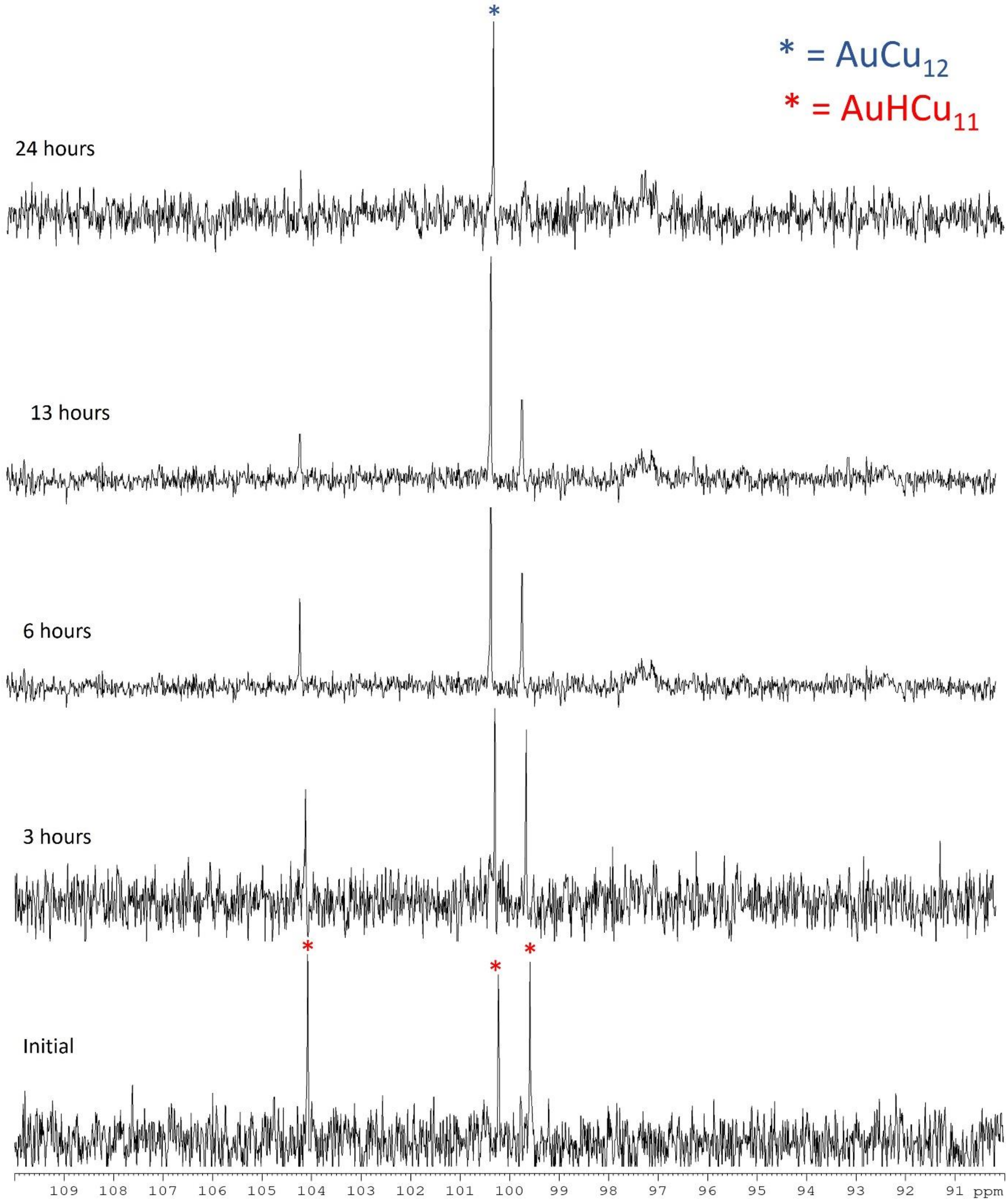


**Figure S27**. Structural transformation of $\mathbf{AuCu_{11}H}$ into $\mathbf{AuCu_{12}}$ in $d_6$-acetone monitored by time-dependent $^{31}P\{^{1}H\}$ NMR spectroscopy.

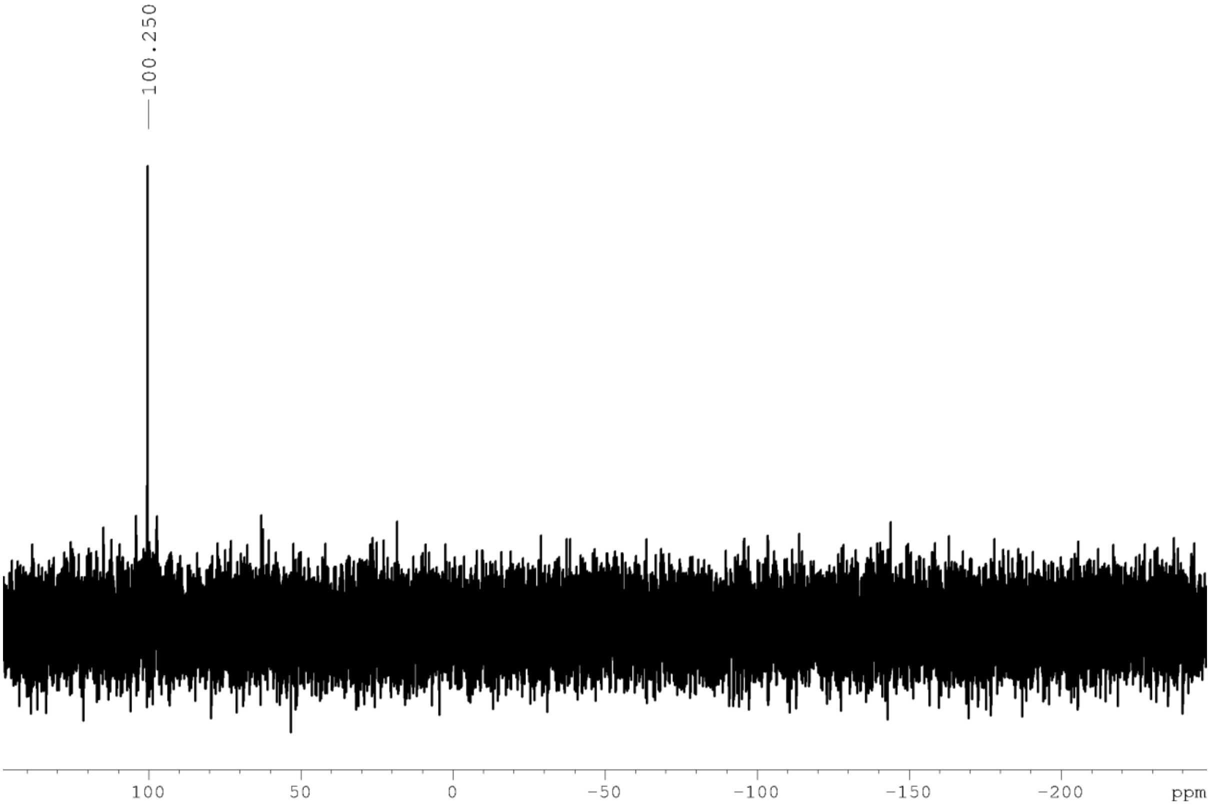


**Figure S28**. $^{31}P\{^1H\}$ NMR spectrum of **AuCu12** in $d_6$-acetone.

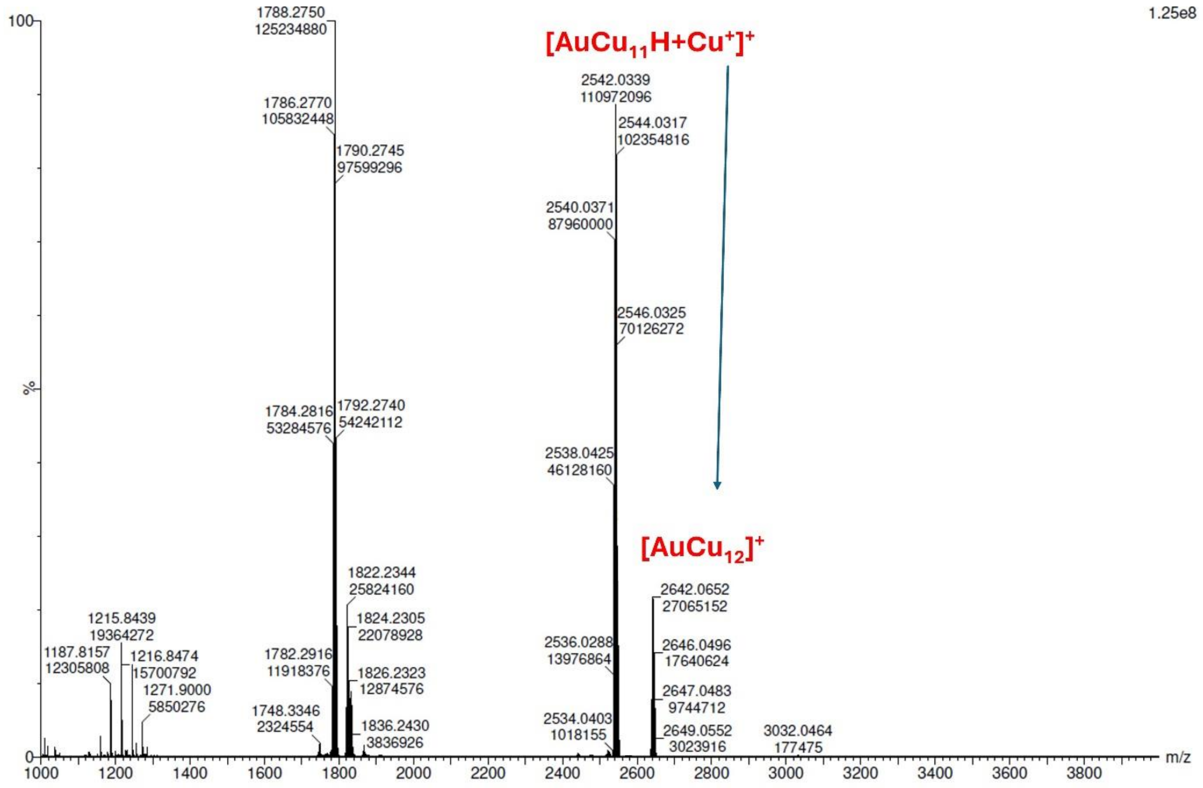


**Figure S29**. Positive-mode ESI-MS spectra of structural transformation of **$AuCu_{11}H$** in acetone.

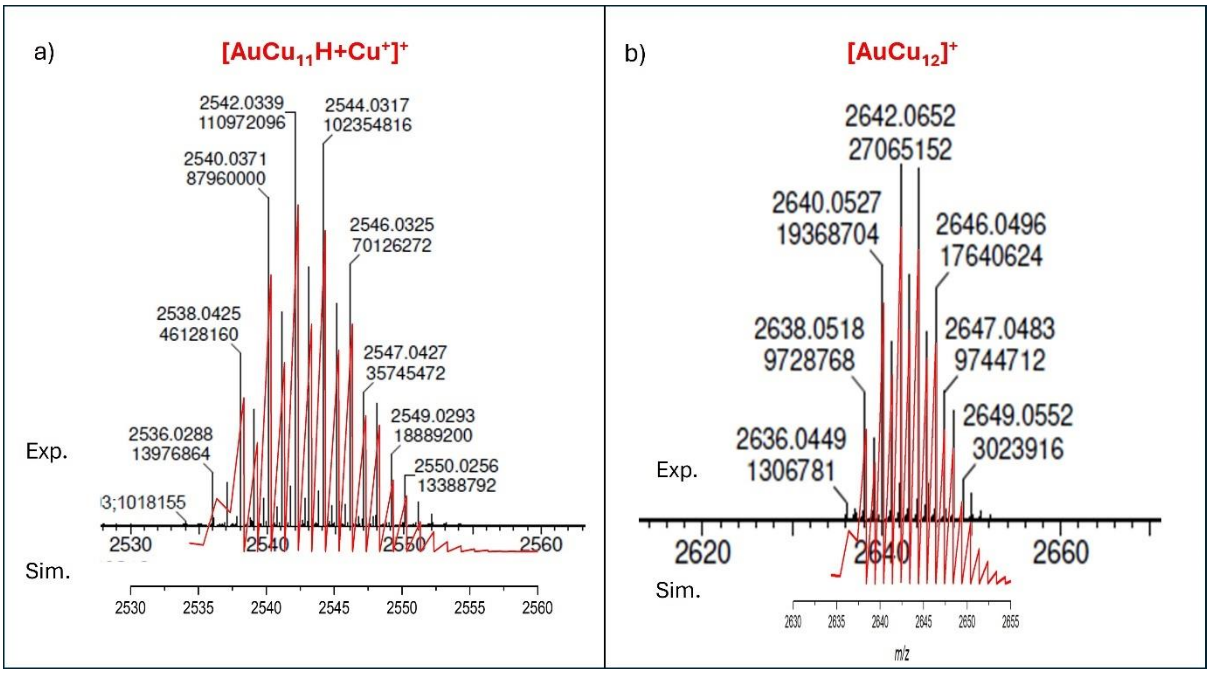


**Figure S30**. Isotope pattern of (a) **$AuCu_{11}H$** and (b) **$AuCu_{12}$** in structural transformation; inset show experimental (black) and simulated (red) isotopic distribution pattern.

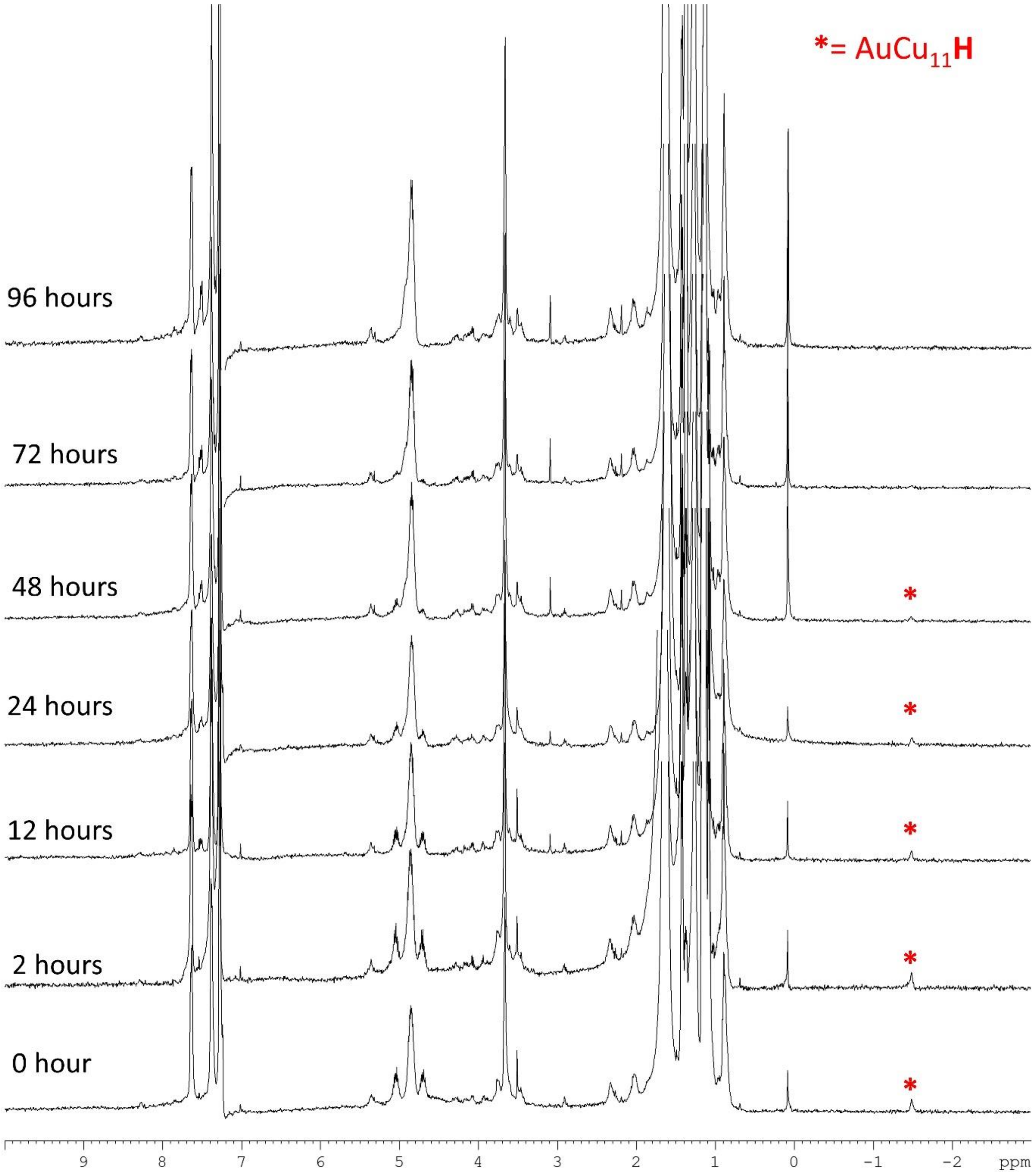

**Figure S31**. Structural transformation of $\mathbf{AuCu_{11}H}$ into $\mathbf{AuCu_{12}}$ via $\mathbf{AuCu_{11}Cl}$ in $CDCl_3$ monitored by time-dependent $^1$H NMR spectroscopy.

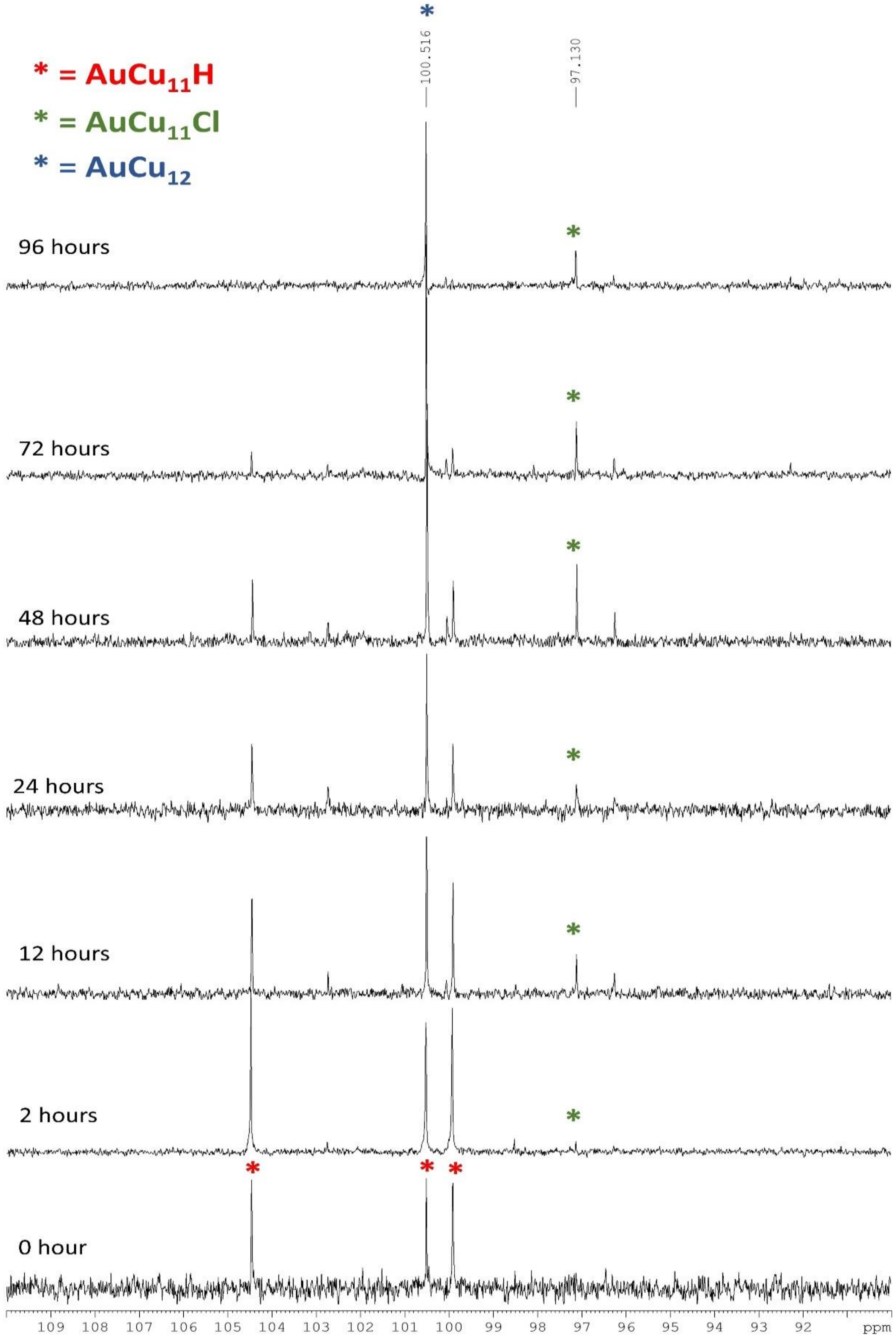


**Figure S32**. Structural transformation of **$AuCu_{11}H$** into **$AuCu_{12}$** via **$AuCu_{11}Cl$** in $CDCl_3$ monitored by time-dependent $^{31}P\{^{1}H\}$ NMR spectroscopy.

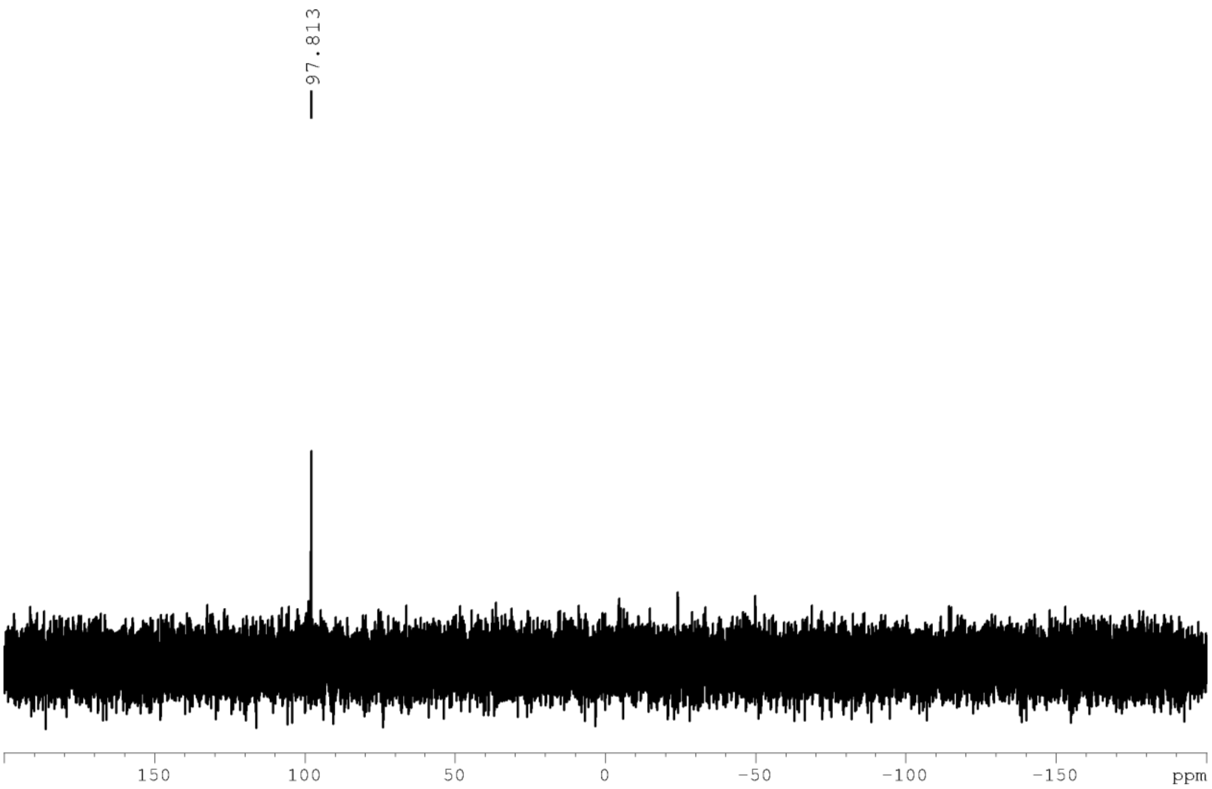

**Figure S33**. $^{31}P\{^{1}H\}$ NMR spectrum of **$AuCu_{11}Cl$** in $CDCl_3$.

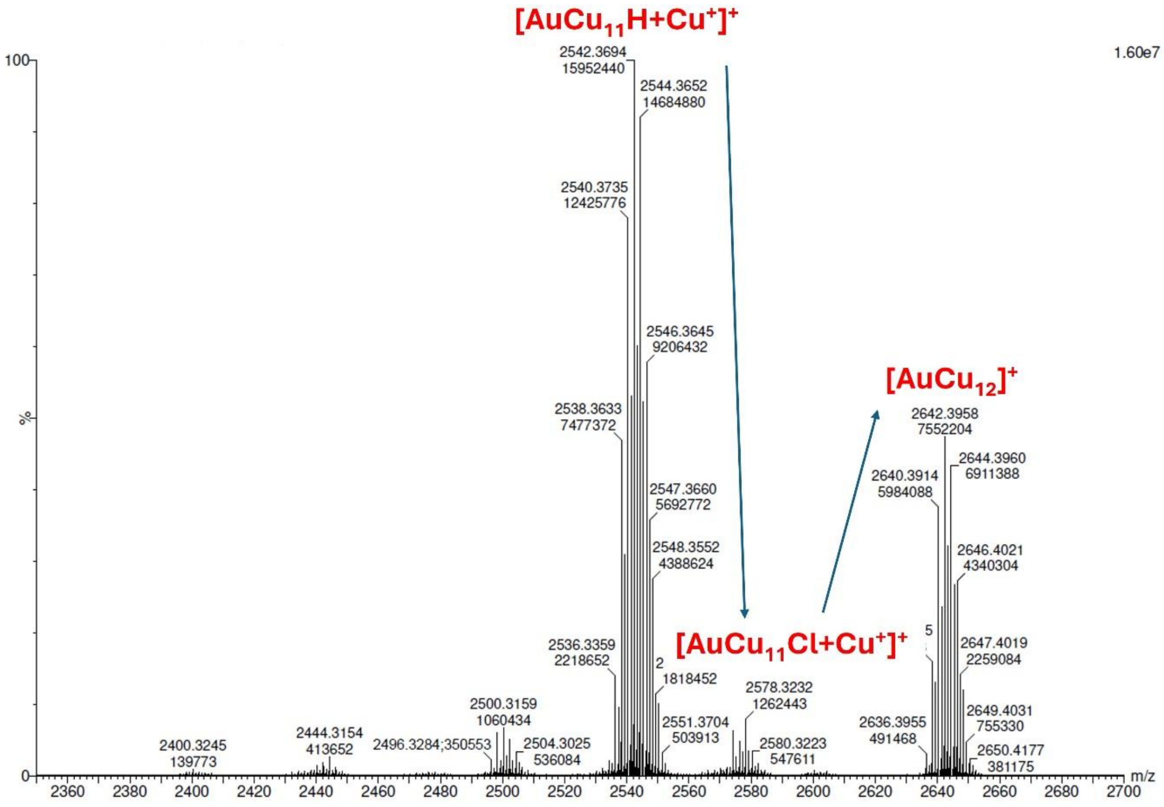


**Figure S34**. Positive-mode ESI-MS spectra of structural transformation of **AuCu$_{11}$H** in $CH_2Cl_2$.

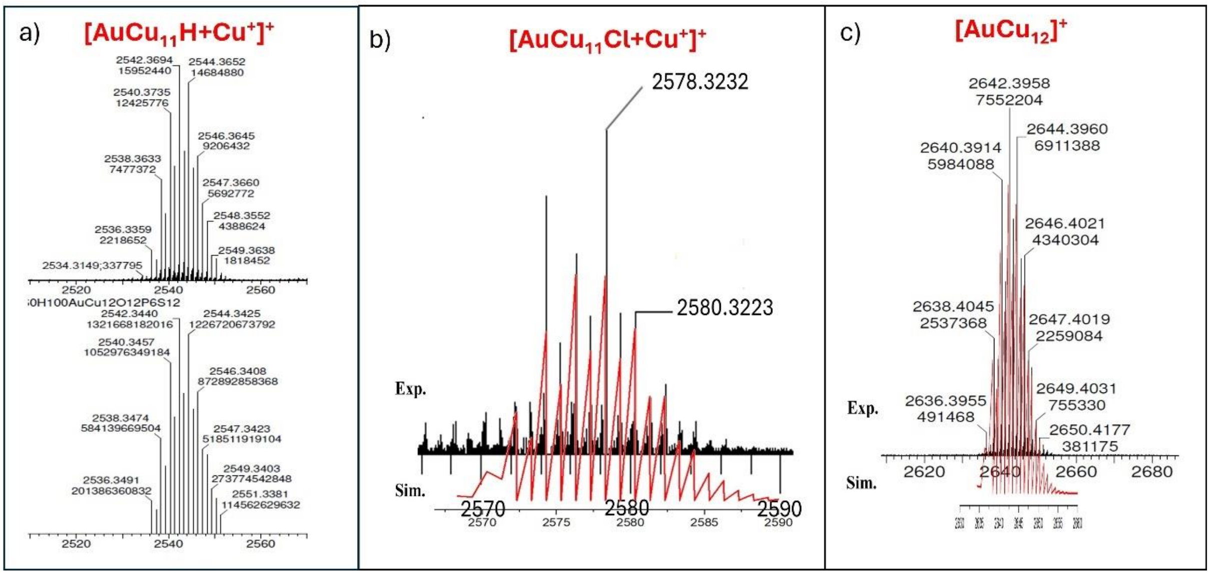


**Figure S35**. Isotope patten of (a) **$AuCu_{11}H$**, (b) **$AuCu_{11}Cl$**, and (c) **$AuCu_{12}$** in structural transformation; inset show experimental (black) and simulated (red) isotopic distribution pattern.

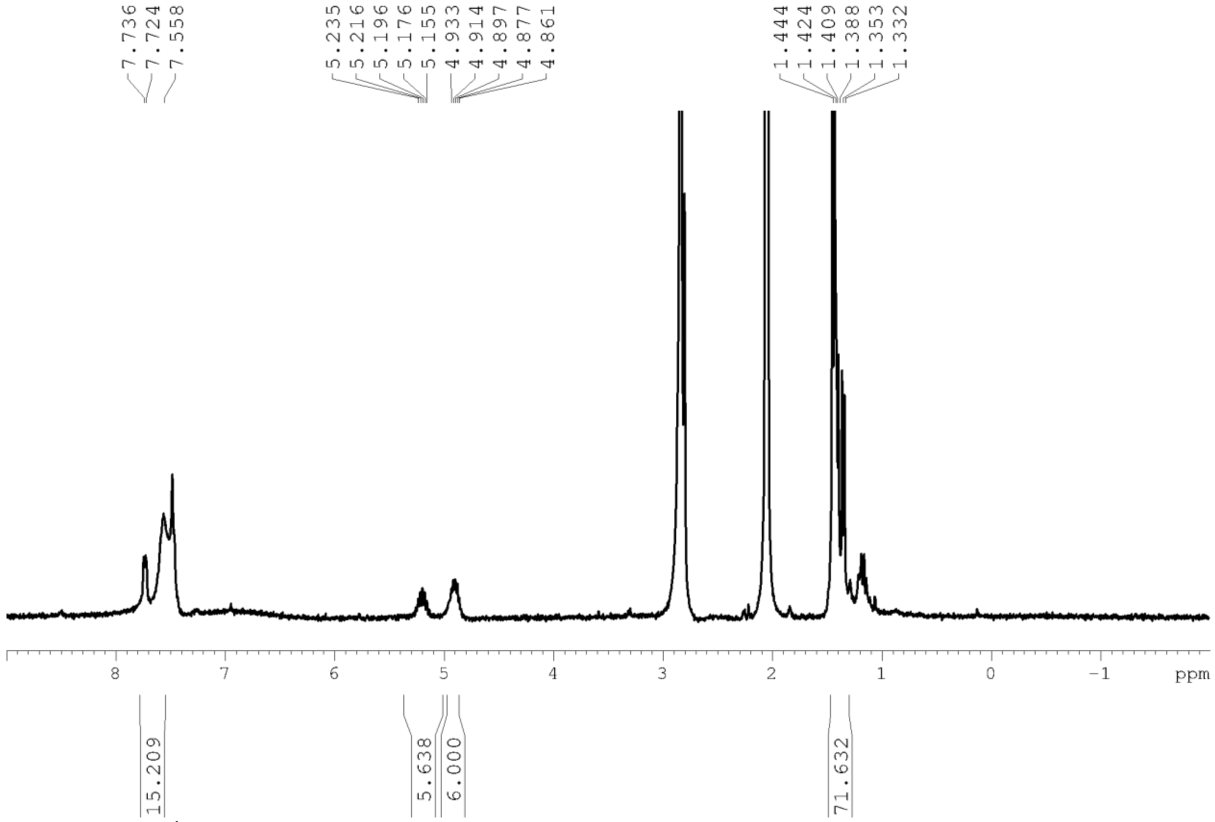


**Figure S36**. $^{1}$H NMR spectrum of **$AuCu_{11}Cl$** in *$d_6$*-acetone.

**Table S1**. 298 K absorption, emission, and lifetime of **$AuCu_{11}H$**, **$AuCu_{11}Cl$**, and **$AuCu_{12}$**.

| **Compound** | **$\lambda_{max}$(nm)** | **$\lambda_{em}$ (nm)** | **Lifetime, (μs)** | **$\Phi_{em}$** | **$k_{obs}$,[a] 1/τ($\mu s^{-1}$)** | **$k_{RAD}$[b] ($\mu s^{-1}$)** | **$k_{NRD}$[c] ($\mu s^{-1}$)** |
|---|---|---|---|---|---|---|---|
| **$AuCu_{11}H$** | 318, 424, 487, 533 | 633 | 1.71 | 0.327 | 0.58 | 0.19 | 0.39 |
| **$AuCu_{11}Cl$** | 328, 432, 485, 540 | 606 | 3.75 | 0.033 | 0.27 | 0.009 | 0.26 |
| **$AuCu_{12}$** | 326, 433, 480, 540 | 637 | 3.20 | 0.32 | 0.31 | 0.10 | 0.21 |

[a]$k_{obs} = k_{RAD} + k_{NRD}$, [b]$k_{RAD} = \Phi_{em}/\tau$, [c]$k_{NRD} = (1-\Phi_{em})/\tau$; $k_{RAD}$ = radiative rate constant, $k_{obs}$= mean excited-state decay rate constant, and $k_{NRD}$= nonradiative rate constant.

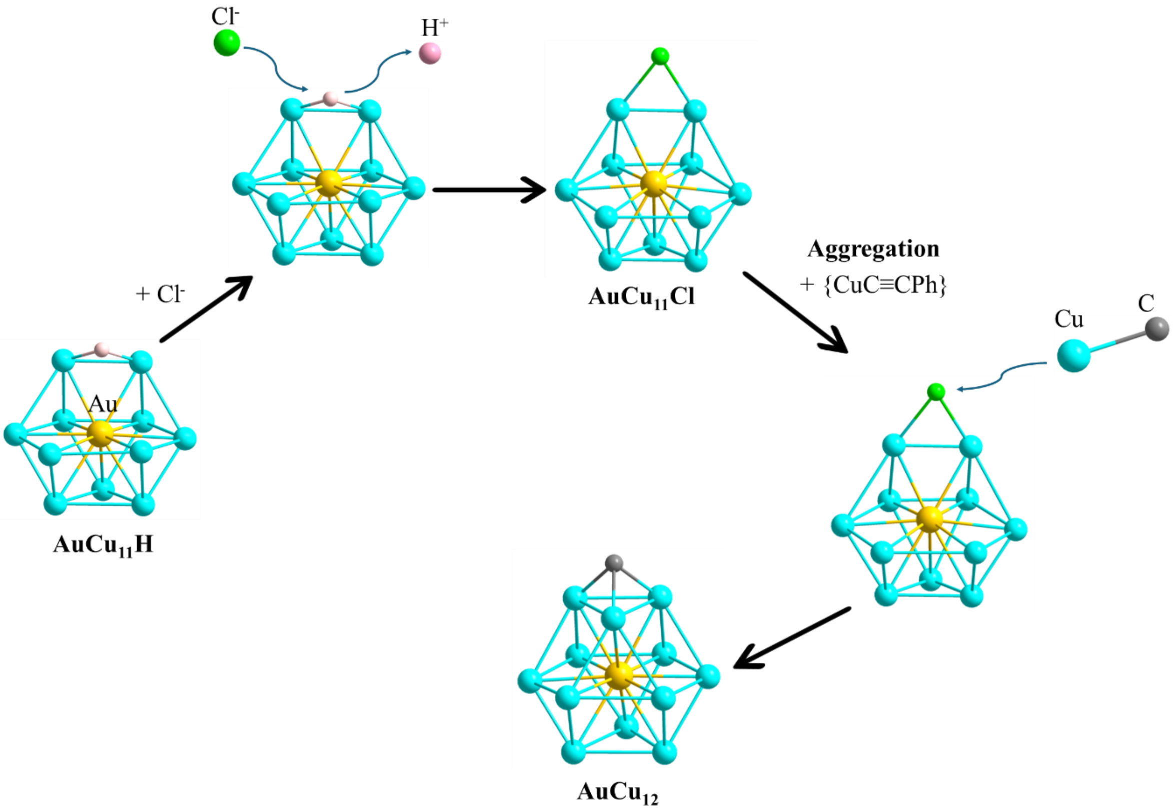


**Scheme S1**. A proposed mechanism of chloride-hydride ligand exchange, and aggregation within the transformation core of **$AuCu_{11}H$** to **$AuCu_{12}$** via **$AuCu_{11}Cl$** in chloride-containing solvent.

**Table S2**. Cartesian coordinates of the DFT-optimezed structure of **$AuCu_{11}H$**.

| | | | |
|---|---|---|---|
| Au | 0.095621 | -0.130302 | 0.333739 |
| Cu1 | -2.543087 | -1.007363 | 0.601874 |
| Cu2 | -1.373752 | -1.050237 | -1.796426 |
| Cu3 | -0.704731 | -2.861573 | -0.048113 |
| Cu4 | -1.877508 | 1.860040 | 0.763614 |
| Cu5 | -0.673009 | 1.639173 | -1.604270 |
| Cu6 | 0.577234 | 2.669368 | 0.478096 |
| Cu7 | 1.352161 | -0.135480 | -2.112966 |
| Cu8 | 2.731650 | 0.705228 | -0.097001 |
| Cu9 | 2.169237 | -1.969732 | -0.259064 |
| Cu10 | 1.422831 | 0.813534 | 2.540724 |
| Cu11 | -0.972821 | -0.008806 | 2.850733 |
| C1 | 3.203727 | -0.655224 | -1.521675 |
| C2 | 4.204344 | -1.309369 | -1.823620 |
| C3 | 5.352022 | -2.072006 | -2.159903 |
| C4 | 5.537875 | -3.342571 | -1.600716 |
| C5 | 6.656799 | -4.089406 | -1.924078 |
| C6 | 7.608175 | -3.589262 | -2.803399 |
| C7 | 7.434043 | -2.329618 | -3.361458 |
| C8 | 6.317450 | -1.576065 | -3.045007 |
| H1 | 4.798184 | -3.724572 | -0.907825 |
| H2 | 6.787672 | -5.071029 | -1.482998 |
| H3 | 8.483276 | -4.178487 | -3.052622 |
| H4 | 8.173316 | -1.932070 | -4.047842 |
| H5 | 6.179907 | -0.593493 | -3.479729 |
| C9 | -1.116415 | 3.324141 | -0.410874 |
| C10 | -1.535832 | 4.253165 | -1.101329 |
| C11 | -2.014162 | 5.294023 | -1.936689 |
| C12 | -2.423111 | 6.524254 | -1.406676 |
| C13 | -2.887854 | 7.528303 | -2.238210 |
| C14 | -2.953105 | 7.325608 | -3.610369 |
| C15 | -2.549029 | 6.109333 | -4.145830 |

| | | | |
|---|---|---|---|
| C16 | -2.083341 | 5.099650 | -3.322429 |
| H6 | -2.370129 | 6.681113 | -0.336035 |
| H7 | -3.200645 | 8.475538 | -1.813297 |
| H8 | -3.316543 | 8.113522 | -4.260115 |
| H9 | -2.595937 | 5.945482 | -5.216567 |
| H10 | -1.765827 | 4.149504 | -3.734009 |
| C17 | -2.514336 | -2.430249 | -0.900338 |
| C18 | -3.509949 | -3.154828 | -0.913096 |
| C19 | -4.653059 | -3.994055 | -0.920026 |
| C20 | -4.517781 | -5.385202 | -0.828807 |
| C21 | -5.637557 | -6.198546 | -0.836898 |
| C22 | -6.907450 | -5.644031 | -0.927943 |
| C23 | -7.051916 | -4.264821 | -1.003651 |
| C24 | -5.938770 | -3.442806 | -0.999563 |
| H11 | -3.526880 | -5.811978 | -0.734005 |
| H12 | -5.518530 | -7.273754 | -0.763812 |
| H13 | -7.782071 | -6.284355 | -0.931433 |
| H14 | -8.041971 | -3.826090 | -1.060586 |
| H15 | -6.042837 | -2.365205 | -1.044948 |
| S1 | -3.962992 | 0.846689 | 0.241235 |
| S2 | -2.466600 | 0.782406 | -2.893122 |
| S3 | 0.127179 | -1.558406 | -3.568770 |
| S4 | 1.105681 | -3.850497 | -1.190599 |
| S5 | -0.531826 | -3.931276 | 1.996170 |
| S6 | -2.775695 | -1.455175 | 2.871734 |
| S7 | -2.133591 | 2.037187 | 3.105004 |
| S8 | 1.122077 | 3.147548 | 2.729016 |
| S9 | 0.974202 | 1.927902 | -3.295610 |
| S10 | 2.691672 | 3.034428 | -0.524038 |
| S11 | 2.989242 | -2.664259 | 1.790644 |
| S12 | 3.684209 | 0.694761 | 2.042232 |
| P1 | -4.089862 | 1.051247 | -1.752471 |
| P2 | 0.896394 | -3.371518 | -3.127441 |
| P3 | -2.075059 | -3.362462 | 3.074776 |

| | | | |
|---|---|---|---|
| P4 | -0.622656 | 3.188278 | 3.745478 |
| P5 | 2.208758 | 3.269436 | -2.471195 |
| P6 | 4.277380 | -1.250620 | 2.243106 |
| O1 | -5.249065 | 0.043490 | -2.173441 |
| O2 | -4.600782 | 2.503969 | -2.141220 |
| O3 | 0.062208 | -4.483768 | -3.892996 |
| O4 | 2.312691 | -3.549955 | -3.795048 |
| O5 | -3.294570 | -4.344762 | 2.846087 |
| O6 | -1.797225 | -3.596052 | 4.626347 |
| O7 | -0.398438 | 2.798195 | 5.257281 |
| O8 | -1.089961 | 4.701641 | 3.887366 |
| O9 | 3.547692 | 3.391117 | -3.317748 |
| O10 | 1.576946 | 4.693313 | -2.713539 |
| O11 | 4.810966 | -1.416117 | 3.737739 |
| O12 | 5.624205 | -1.441632 | 1.436276 |
| C25 | -5.666292 | -0.108507 | -3.558715 |
| H16 | -5.295723 | 0.755636 | -4.120446 |
| C26 | -5.075787 | -1.378063 | -4.127667 |
| H17 | -3.987845 | -1.360125 | -4.072524 |
| H18 | -5.369869 | -1.478894 | -5.175431 |
| H19 | -5.440696 | -2.249484 | -3.580449 |
| C27 | -7.177949 | -0.116718 | -3.563839 |
| H20 | -7.584492 | 0.798502 | -3.131116 |
| H21 | -7.554068 | -0.966783 | -2.990040 |
| H22 | -7.545321 | -0.207063 | -4.588499 |
| C28 | -5.799599 | 3.096376 | -1.573565 |
| H23 | -6.398269 | 2.293699 | -1.129340 |
| C29 | -5.410674 | 4.095766 | -0.509140 |
| H24 | -4.835796 | 3.617707 | 0.283719 |
| H25 | -6.311910 | 4.531566 | -0.070692 |
| H26 | -4.812207 | 4.898146 | -0.944845 |
| C30 | -6.552031 | 3.734722 | -2.718810 |
| H27 | -7.472721 | 4.192816 | -2.350504 |
| H28 | -6.813561 | 3.001502 | -3.483287 |

| | | | |
|---|---|---|---|
| H29 | -5.939691 | 4.512126 | -3.181217 |
| C31 | -1.352500 | -4.717531 | -3.665524 |
| H30 | -1.685484 | -4.062867 | -2.854874 |
| C32 | -1.504378 | -6.162999 | -3.254652 |
| H31 | -1.119816 | -6.823073 | -4.036168 |
| H32 | -2.559558 | -6.394757 | -3.095080 |
| H33 | -0.958793 | -6.361129 | -2.330697 |
| C33 | -2.093781 | -4.372333 | -4.935452 |
| H34 | -1.755430 | -5.004181 | -5.760759 |
| H35 | -1.933607 | -3.326791 | -5.202809 |
| H36 | -3.164946 | -4.532537 | -4.793500 |
| C34 | 2.489536 | -3.423088 | -5.234516 |
| H37 | 1.515969 | -3.191382 | -5.677676 |
| C35 | 2.982262 | -4.755267 | -5.747982 |
| H38 | 3.933432 | -5.013246 | -5.276915 |
| H39 | 3.133288 | -4.705082 | -6.828939 |
| H40 | 2.259490 | -5.543205 | -5.532948 |
| C36 | 3.445786 | -2.284109 | -5.490332 |
| H41 | 3.059281 | -1.356479 | -5.066080 |
| H42 | 3.577171 | -2.148494 | -6.566705 |
| H43 | 4.419427 | -2.495128 | -5.043785 |
| C37 | -4.495229 | -4.275586 | 3.661305 |
| H44 | -4.386326 | -3.443184 | 4.363136 |
| C38 | -5.668057 | -4.014942 | 2.747904 |
| H45 | -5.763389 | -4.810973 | 2.006230 |
| H46 | -5.544507 | -3.065737 | 2.225595 |
| H47 | -6.590295 | -3.971658 | 3.332666 |
| C39 | -4.601294 | -5.575171 | 4.425254 |
| H48 | -4.674936 | -6.416859 | 3.732660 |
| H49 | -5.493677 | -5.566796 | 5.055815 |
| H50 | -3.726598 | -5.720531 | 5.060233 |
| C40 | -0.671938 | -2.992120 | 5.309151 |
| H51 | -0.093235 | -2.418538 | 4.577001 |
| C41 | -1.211052 | -2.065668 | 6.374258 |

| | | | |
|---|---|---|---|
| H52 | -1.824801 | -1.280980 | 5.929595 |
| H53 | -0.383934 | -1.592800 | 6.909169 |
| H54 | -1.815943 | -2.621537 | 7.095320 |
| C42 | 0.173698 | -4.116925 | 5.858851 |
| H55 | 1.029625 | -3.712845 | 6.403352 |
| H56 | 0.544227 | -4.747128 | 5.049278 |
| H57 | -0.412839 | -4.732900 | 6.545334 |
| C43 | 0.604785 | 3.442253 | 6.086063 |
| H58 | 1.198065 | 4.107711 | 5.450639 |
| C44 | 1.490488 | 2.353753 | 6.641915 |
| H59 | 0.901085 | 1.654777 | 7.240187 |
| H60 | 2.265835 | 2.788528 | 7.276946 |
| H61 | 1.971127 | 1.803188 | 5.832454 |
| C45 | -0.111917 | 4.243635 | 7.147363 |
| H62 | -0.750531 | 5.001767 | 6.692924 |
| H63 | 0.615588 | 4.740986 | 7.793167 |
| H64 | -0.731305 | 3.588641 | 7.764327 |
| C46 | -1.446159 | 5.507108 | 2.734399 |
| H65 | -1.328072 | 4.895744 | 1.833774 |
| C47 | -0.496474 | 6.680668 | 2.680735 |
| H66 | -0.730839 | 7.314576 | 1.822277 |
| H67 | 0.534221 | 6.336772 | 2.582401 |
| H68 | -0.582251 | 7.284002 | 3.588023 |
| C48 | -2.894767 | 5.906316 | 2.893545 |
| H69 | -3.032134 | 6.487563 | 3.808674 |
| H70 | -3.533521 | 5.023272 | 2.940269 |
| H71 | -3.213082 | 6.518175 | 2.046317 |
| C49 | 4.503954 | 2.306540 | -3.434608 |
| H72 | 4.132606 | 1.453259 | -2.858785 |
| C50 | 4.587672 | 1.938399 | -4.897598 |
| H73 | 3.613083 | 1.617983 | -5.268407 |
| H74 | 5.297560 | 1.120723 | -5.041799 |
| H75 | 4.924114 | 2.794256 | -5.488054 |
| C51 | 5.814068 | 2.783841 | -2.853546 |

| | | | |
|---|---|---|---|
| H76 | 6.180942 | 3.655432 | -3.401596 |
| H77 | 6.565175 | 1.993414 | -2.920661 |
| H78 | 5.693747 | 3.052977 | -1.803123 |
| C52 | 2.295151 | 5.908768 | -2.357622 |
| H79 | 3.253909 | 5.620205 | -1.915618 |
| C53 | 2.530954 | 6.684697 | -3.631859 |
| H80 | 3.122261 | 6.099012 | -4.336697 |
| H81 | 3.067601 | 7.610295 | -3.410399 |
| H82 | 1.577897 | 6.940797 | -4.100240 |
| C54 | 1.472760 | 6.656817 | -1.337057 |
| H83 | 0.506566 | 6.941619 | -1.758217 |
| H84 | 2.001193 | 7.563261 | -1.031604 |
| H85 | 1.301738 | 6.038626 | -0.454716 |
| C55 | 3.939992 | -1.307849 | 4.885569 |
| H86 | 2.930282 | -1.069840 | 4.531852 |
| C56 | 3.932990 | -2.647186 | 5.585317 |
| H87 | 4.945880 | -2.919495 | 5.892264 |
| H88 | 3.545905 | -3.423620 | 4.924370 |
| H89 | 3.304694 | -2.600837 | 6.477930 |
| C57 | 4.459844 | -0.183054 | 5.751033 |
| H90 | 5.481153 | -0.396256 | 6.076734 |
| H91 | 3.832510 | -0.069266 | 6.638108 |
| H92 | 4.457680 | 0.758724 | 5.199926 |
| C58 | 6.790371 | -0.612877 | 1.683708 |
| H93 | 6.527592 | 0.131098 | 2.442083 |
| C59 | 7.149185 | 0.086719 | 0.394791 |
| H94 | 8.015939 | 0.733276 | 0.553086 |
| H95 | 6.315758 | 0.699571 | 0.048472 |
| H96 | 7.394557 | -0.641806 | -0.380796 |
| C60 | 7.886659 | -1.511859 | 2.206693 |
| H97 | 7.575955 | -1.999146 | 3.131599 |
| H98 | 8.787728 | -0.926422 | 2.405296 |
| H99 | 8.128676 | -2.280757 | 1.469327 |
| H100 | 0.573722 | -0.453601 | 3.125499 |

202

scf done: ************

| | | | |
|---|---|---|---|
| Au | 0.085618 | -0.034352 | 0.261218 |
| Cu | -2.065689 | -2.000468 | 0.676718 |
| Cu | -0.968069 | -1.736219 | -1.885585 |
| Cu | 0.417774 | -2.845302 | -0.045194 |
| Cu | -2.373123 | 1.234931 | 0.734250 |
| Cu | -1.366626 | 1.458992 | -1.662982 |
| Cu | -0.460255 | 2.693157 | 0.379616 |
| Cu | 1.409896 | 0.366213 | -2.264067 |
| Cu | 2.340517 | 1.659117 | -0.160986 |
| Cu | 2.683178 | -1.307658 | -0.185118 |
| Cu | 0.957193 | 1.609882 | 2.134186 |
| Cu | -1.221887 | -0.136210 | 2.543186 |
| C | 3.206272 | 0.370117 | -1.416823 |
| C | 4.305106 | -0.166271 | -1.608368 |
| C | 5.606549 | -0.640484 | -1.924916 |
| C | 6.106283 | -1.820934 | -1.366272 |
| C | 7.378190 | -2.260120 | -1.689263 |
| C | 8.172040 | -1.536271 | -2.568182 |
| C | 7.685379 | -0.361488 | -3.127295 |
| C | 6.414817 | 0.084376 | -2.810910 |
| H | 5.486601 | -2.380019 | -0.676953 |
| H | 7.752578 | -3.176967 | -1.248512 |
| H | 9.167736 | -1.885351 | -2.817162 |
| H | 8.300010 | 0.209670 | -3.814219 |
| H | 6.030652 | 0.999675 | -3.245500 |
| C | -2.332883 | 2.951317 | -0.435341 |
| C | -3.021728 | 3.556974 | -1.258993 |
| C | -3.817730 | 4.272766 | -2.190402 |
| C | -4.661037 | 5.309565 | -1.768746 |
| C | -5.435944 | 6.002740 | -2.682322 |
| C | -5.385604 | 5.678376 | -4.031566 |
| C | -4.551847 | 4.653858 | -4.460616 |

| | | | |
|---|---|---|---|
| C | -3.774248 | 3.955016 | -3.554062 |
| H | -4.697344 | 5.562792 | -0.715671 |
| H | -6.083091 | 6.802426 | -2.339581 |
| H | -5.992487 | 6.223084 | -4.745725 |
| H | -4.505582 | 4.396767 | -5.512941 |
| H | -3.121544 | 3.156489 | -3.885277 |
| C | -1.412808 | -3.340212 | -0.777699 |
| C | -2.246758 | -4.239996 | -0.649490 |
| C | -3.138640 | -5.333775 | -0.497699 |
| C | -2.644757 | -6.644016 | -0.459590 |
| C | -3.511212 | -7.712639 | -0.311006 |
| C | -4.877811 | -7.496365 | -0.192584 |
| C | -5.375169 | -6.200029 | -0.218883 |
| C | -4.517639 | -5.124266 | -0.369582 |
| H | -1.575808 | -6.804355 | -0.530730 |
| H | -3.116373 | -8.721882 | -0.280121 |
| H | -5.553076 | -8.336020 | -0.073653 |
| H | -6.440504 | -6.025940 | -0.115616 |
| H | -4.897045 | -4.109408 | -0.386459 |
| S | -3.910124 | -0.564711 | 0.398035 |
| S | -2.540441 | -0.235439 | -2.760832 |
| S | 0.698113 | -1.503000 | -3.500972 |
| S | 2.484718 | -3.507206 | -1.315251 |
| S | 0.835344 | -4.272960 | 1.853237 |
| S | -1.817553 | -2.297092 | 2.975824 |
| S | -2.817121 | 1.442967 | 3.156088 |
| S | -0.139103 | 3.585918 | 2.723813 |
| S | 0.200712 | 2.097480 | -3.291627 |
| S | 1.434230 | 3.814757 | -0.564856 |
| S | 3.876531 | -1.503157 | 1.901861 |
| S | 3.231116 | 1.900945 | 2.035948 |
| P | -4.153648 | -0.501622 | -1.589030 |
| P | 2.034064 | -3.014439 | -3.178390 |
| P | -0.550361 | -3.905693 | 3.180740 |

| | | | |
|---|---|---|---|
| P | -1.772614 | 3.040872 | 3.757554 |
| P | 0.945326 | 3.799687 | -2.518179 |
| P | 4.520711 | 0.315291 | 2.231226 |
| O | -4.891108 | -1.864159 | -1.944229 |
| O | -5.129663 | 0.670501 | -2.020408 |
| O | 1.516289 | -4.267646 | -4.008112 |
| O | 3.372298 | -2.676666 | -3.942026 |
| O | -1.496285 | -5.166711 | 3.316624 |
| O | 0.035271 | -3.810705 | 4.661664 |
| O | -1.421428 | 2.779465 | 5.275572 |
| O | -2.734239 | 4.300851 | 3.869977 |
| O | 2.189098 | 4.293549 | -3.373052 |
| O | -0.112541 | 4.919405 | -2.844467 |
| O | 5.152076 | 0.465203 | 3.692141 |
| O | 5.790443 | 0.632866 | 1.345256 |
| C | -5.266718 | -2.215568 | -3.305432 |
| H | -5.213087 | -1.308019 | -3.916141 |
| C | -4.302792 | -3.250240 | -3.837101 |
| H | -3.282952 | -2.866825 | -3.844656 |
| H | -4.580703 | -3.516905 | -4.859789 |
| H | -4.333919 | -4.151630 | -3.221746 |
| C | -6.690533 | -2.720144 | -3.252163 |
| H | -7.366097 | -1.967936 | -2.842307 |
| H | -6.750721 | -3.616230 | -2.630419 |
| H | -7.030717 | -2.977713 | -4.257593 |
| C | -6.449603 | 0.855987 | -1.438476 |
| H | -6.741162 | -0.082872 | -0.955106 |
| C | -6.398337 | 1.966482 | -0.415500 |
| H | -5.685308 | 1.739328 | 0.376595 |
| H | -7.387052 | 2.093331 | 0.032595 |
| H | -6.107885 | 2.904908 | -0.891136 |
| C | -7.385729 | 1.166020 | -2.583867 |
| H | -8.400562 | 1.309209 | -2.206156 |
| H | -7.401532 | 0.358372 | -3.317350 |

| | | | |
|---|---|---|---|
| H | -7.070845 | 2.083288 | -3.086391 |
| C | 0.303804 | -4.987940 | -3.666351 |
| H | -0.181375 | -4.473128 | -2.831685 |
| C | 0.698204 | -6.382159 | -3.239176 |
| H | 1.223821 | -6.895796 | -4.048134 |
| H | -0.194899 | -6.958583 | -2.986723 |
| H | 1.348537 | -6.345814 | -2.364229 |
| C | -0.599767 | -4.960785 | -4.876382 |
| H | -0.117469 | -5.451459 | -5.725712 |
| H | -0.839836 | -3.933495 | -5.155170 |
| H | -1.531200 | -5.487291 | -4.656213 |
| C | 3.399527 | -2.416242 | -5.371342 |
| H | 2.367936 | -2.406790 | -5.736740 |
| C | 4.168365 | -3.538479 | -6.028655 |
| H | 5.187947 | -3.577462 | -5.638572 |
| H | 4.215915 | -3.375782 | -7.108110 |
| H | 3.684914 | -4.497836 | -5.841448 |
| C | 4.022196 | -1.056876 | -5.577723 |
| H | 3.442171 | -0.288675 | -5.064405 |
| H | 4.050355 | -0.819236 | -6.643895 |
| H | 5.042482 | -1.043074 | -5.188543 |
| C | -2.537658 | -5.275814 | 4.319948 |
| H | -2.585733 | -4.332328 | 4.871930 |
| C | -3.843762 | -5.507751 | 3.598465 |
| H | -3.788177 | -6.412936 | 2.989283 |
| H | -4.075687 | -4.665146 | 2.946128 |
| H | -4.655197 | -5.622340 | 4.321332 |
| C | -2.157108 | -6.399069 | 5.256883 |
| H | -2.073947 | -7.339788 | 4.707633 |
| H | -2.920202 | -6.515858 | 6.030242 |
| H | -1.201213 | -6.190544 | 5.738790 |
| C | 1.105913 | -2.897040 | 4.993778 |
| H | 1.378471 | -2.340480 | 4.089965 |
| C | 0.595943 | -1.949081 | 6.055439 |

| | | | |
|---|---|---|---|
| H | -0.270921 | -1.392738 | 5.695164 |
| H | 1.377775 | -1.235828 | 6.328240 |
| H | 0.306958 | -2.501165 | 6.953329 |
| C | 2.286417 | -3.727255 | 5.439720 |
| H | 3.119783 | -3.079749 | 5.718198 |
| H | 2.613863 | -4.383530 | 4.632585 |
| H | 2.015164 | -4.337432 | 6.305118 |
| C | -0.706225 | 3.753647 | 6.081016 |
| H | -0.413032 | 4.585109 | 5.432163 |
| C | 0.530256 | 3.073938 | 6.618334 |
| H | 0.253849 | 2.206023 | 7.221891 |
| H | 1.096913 | 3.766624 | 7.244836 |
| H | 1.171562 | 2.744376 | 5.799816 |
| C | -1.646247 | 4.243739 | 7.157219 |
| H | -2.528749 | 4.707619 | 6.715328 |
| H | -1.141721 | 4.981484 | 7.785602 |
| H | -1.967599 | 3.412659 | 7.789175 |
| C | -3.321406 | 4.907859 | 2.686832 |
| H | -2.984116 | 4.349135 | 1.807496 |
| C | -2.827437 | 6.332748 | 2.604240 |
| H | -3.249840 | 6.823261 | 1.724072 |
| H | -1.739887 | 6.358884 | 2.523050 |
| H | -3.129681 | 6.895500 | 3.491165 |
| C | -4.822212 | 4.794999 | 2.816971 |
| H | -5.170096 | 5.320800 | 3.709618 |
| H | -5.125011 | 3.749334 | 2.885391 |
| H | -5.307440 | 5.238569 | 1.944356 |
| C | 3.460435 | 3.595294 | -3.406574 |
| H | 3.383238 | 2.706076 | -2.772801 |
| C | 3.710398 | 3.181959 | -4.837990 |
| H | 2.913096 | 2.528699 | -5.195177 |
| H | 4.658201 | 2.644045 | -4.913277 |
| H | 3.759945 | 4.060355 | -5.486160 |
| C | 4.513169 | 4.526738 | -2.854056 |

| | | | |
|---|---|---|---|
| H | 4.576817 | 5.433796 | -3.460444 |
| H | 5.489075 | 4.036013 | -2.863543 |
| H | 4.279344 | 4.806198 | -1.825698 |
| C | 0.127583 | 6.315363 | -2.506941 |
| H | 1.107537 | 6.384096 | -2.024077 |
| C | 0.135273 | 7.096889 | -3.799294 |
| H | 0.920517 | 6.736080 | -4.464775 |
| H | 0.311490 | 8.155205 | -3.592955 |
| H | -0.827294 | 6.998760 | -4.306263 |
| C | -0.945119 | 6.752146 | -1.539605 |
| H | -1.931372 | 6.671354 | -2.001212 |
| H | -0.778516 | 7.792805 | -1.250694 |
| H | -0.928202 | 6.132130 | -0.642394 |
| C | 4.392161 | 0.245023 | 4.896687 |
| H | 3.387309 | -0.088322 | 4.613522 |
| C | 5.088056 | -0.847953 | 5.675370 |
| H | 6.104358 | -0.539003 | 5.931886 |
| H | 5.142198 | -1.763253 | 5.084549 |
| H | 4.548872 | -1.056291 | 6.602733 |
| C | 4.306400 | 1.552472 | 5.651379 |
| H | 5.306861 | 1.899642 | 5.921415 |
| H | 3.729190 | 1.420211 | 6.569738 |
| H | 3.823696 | 2.317580 | 5.041842 |
| C | 6.561434 | 1.852588 | 1.497216 |
| H | 6.074809 | 2.474754 | 2.254335 |
| C | 6.555437 | 2.574459 | 0.171144 |
| H | 7.120652 | 3.506256 | 0.252347 |
| H | 5.533698 | 2.811228 | -0.128592 |
| H | 7.013652 | 1.953241 | -0.601510 |
| C | 7.944753 | 1.462965 | 1.964459 |
| H | 7.894522 | 0.939274 | 2.919770 |
| H | 8.563466 | 2.355334 | 2.087004 |
| H | 8.421161 | 0.808343 | 1.231040 |
| H | 1.156006 | -1.471817 | 0.584017 |
